# Top-Down Proteomic Profiling of Protein Corona by High-Throughput Capillary Isoelectric Focusing-Mass Spectrometry

**DOI:** 10.1021/jasms.4c00463

**Published:** 2025-03-02

**Authors:** Reyhane Tabatabaeian Nimavard, Seyed Amirhossein Sadeghi, Morteza Mahmoudi, Guijie Zhu, Liangliang Sun

**Affiliations:** Department of Chemistry, Michigan State University, East Lansing, Michigan 48824, United States; Department of Chemistry, Michigan State University, East Lansing, Michigan 48824, United States; Precision Health Program and Department of Radiology, College of Human Medicine, Michigan State University, East Lansing, Michigan 48824, United States; Department of Chemistry, Michigan State University, East Lansing, Michigan 48824, United States; Department of Chemistry, Michigan State University, East Lansing, Michigan 48824, United States

**Keywords:** top-down proteomics, protein corona, cIEF-MS/MS, proteoform, nanomedicine

## Abstract

In the rapidly evolving field of nanomedicine, understanding the interactions between nanoparticles (NPs) and biological systems is crucial. A pivotal aspect of these interactions is the formation of a protein corona when NPs are exposed to biological fluids (e.g., human plasma), which significantly influences their behavior and functionality. This study introduces an advanced capillary isoelectric focusing tandem mass spectrometry (cIEF-MS/MS) platform designed to enable high-throughput and reproducible top-down proteomic analysis of protein corona. Our cIEF-MS/MS technique completed each analysis within 30 min. It produced reproducible proteoform measurements of protein corona for at least 50 runs regarding the proteoforms’ migration time [relative standard deviations (RSDs) <4%], the proteoforms’ intensity (Pearson’s correlation coefficients between any two runs >0.90), the number of proteoform identifications (71 ± 10), and the number of proteoform-spectrum matches (PrSMs) (196 ± 30). Of the 53 identified genes, 33 are potential biomarkers of various diseases (e.g., cancer, cardiovascular disease, and Alzheimer’s disease). We identified 1−102 proteoforms per potential protein biomarker, containing various sequence variations or post-translational modifications. Delineating proteoforms in protein corona by our cIEF-MS/MS in a reproducible and high-throughput fashion will benefit our understanding of nanobiointeractions and advance both diagnostic and therapeutic nanomedicine technologies.

## Graphical Abstract

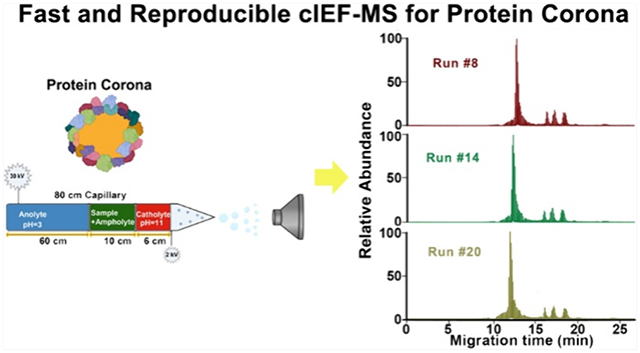

## INTRODUCTION

In the past decade, extensive research has been conducted in the field of nanomedicine to achieve safer designs and more efficient therapeutic and diagnostic outcomes.^1,2^ To accomplish this, a deep understanding of the protein corona (a layer of biomolecules that adheres to the surfaces of nanoparticles (NPs) upon their interaction with biological fluids^3–7^) is essential. Comprehensive understanding of protein corona composition can substantially improve the capability of nanomedicine community to predict the way NPs interact with biosystems which in turn enables them to design safer and more efficient nanomedicines for both therapeutic and diagnostic (e.g., discovering novel protein biomarkers of diseases from human plasma) purposes.^8–11^

Mass spectrometry (MS)-based bottom-up proteomics (BUP) has been widely utilized to study the protein corona and generated rich information on the gene products and protein post-translational modifications (PTMs) in the protein corona.^12–14^ However, the BUP approach cannot provide information about the exact forms of protein molecules (“proteoforms”) in the protein corona due to the enzymatic digestion step. Proteoforms from the same gene can have divergent biological functions,^15,16^ and proteoforms in protein corona can significantly influence NP−cell interactions.^17,18^ Therefore, it is crucial to characterize the protein corona in a proteoform-specific manner.

MS-based top-down proteomics (TDP) is ideal for proteoform identification and quantification and has been widely used to study proteoform functions and discover proteoform biomarkers of diseases.^19–22^ We recently presented the first example of MS-based TDP for measuring the proteoforms in protein coronas by capillary zone electrophoresis (CZE)-MS.^23^ We also showed the advantages of capillary isoelectric focusing (cIEF)-MS for TDP of protein corona regarding the improved separation resolution and detection of large proteoforms compared to CZE-MS.^24^ Our previous CE-MS studies of protein corona required at least 1 h per run and did not provide long-term reproducibility investigations of CE-MS for TDP of protein coronas.

To establish a high-throughput, robust, and reproducible workflow for the broad application of MS-based top-down proteomics in protein corona analysis (aimed at advancing nanomedicine and discovering novel proteoform biomarkers of diseases), here we aim to develop an improved cIEF-MS/MS platform that enhances analysis throughput for top-down proteomic analysis of NP protein coronas with excellent reproducibility.

## EXPERIMENTAL WORKFLOW

### Chemicals and Materials.

The following materials were purchased from Sigma-Aldrich (St. Louis, MO): ammonium bicarbonate (ABC), 3-(trimethoxysilyl) propyl methacrylate (*γ*-MAPS), dithiothreitol (DTT), ammonia hydroxide (NH_3_H_2_O), ammonium acetate (NH_4_Ac), ammonium persulfate (APS), Pharmalytes with pI ranges of 3–10, 5–8 and 8–10.5 (GE Healthcare). HPLC-grade acetic acid (AA), MS-grade water, methanol (MeOH), formic acid (FA), Amicon Ultra (0.5 mL, 10 kDa cutoff size) centrifugal filter units, and fused silica capillaries (50 *μ*m i.d./360 *μ*m o.d., Polymicro Technologies) were purchased from Fisher Scientific (Pittsburgh, PA). Acrylamide was purchased from Acros Organics (Fair Lawn, NJ). A healthy human plasma sample was purchased from Innovative Research (www.innov-research.com) and diluted to 55% using phosphate buffer solution (PBS, 1X). Polystyrene NPs (PSNPs, ~100 nm) were obtained from Polysciences (www.polysciences.com).

### Sample Preparation and Characterization.

The sample preparation procedure for protein coronas is the same as for ref.^23,25^ Briefly, PSNPs were mixed with 55% human plasma. This mixture was stirred constantly for 1 h at a temperature of 37 °C to allow the formation of a protein corona. After an hour, the protein−NP complexes were separated by centrifugation at 14 000*g* for 20 min to remove unbound proteins. The resulting pellet was then washed twice with cold PBS.

Dynamic light scattering (DLS) analysis was performed to measure the size distribution of PSNPs before and after protein corona formation. The measurements were conducted at room temperature using a Zetasizer Nano Series DLS instrument (Malvern Instruments) equipped with a helium−neon laser at a wavelength of 632 nm.

For the collected protein corona coated PSNPS, the proteins were extracted from the NP surface by incubating the pellet in a 0.4% SDS solution with agitation for 1.5 h at 60 °C, and the extracted protein corona-containing supernatant was separated by centrifugation. An Amicon Ultra centrifugal filter with a 10 kDa molecular weight cutoff was used to exchange the buffer and remove the SDS. Finally, the protein corona sample in 100 mM ammonium bicarbonate (NH_4_HCO_3_) was measured using a BCA assay to determine the protein concentration, and it was adjusted to 1.5 mg/mL for MS analysis.

### cIEF-MS/MS Analysis.

An automated cIEF-MS/MS system was built by combining a CESI 8000 Plus CE system (Beckman Coulter) with an Orbitrap Exploris 480 mass spectrometer (Thermo Fisher Scientific) using an in-house electrokinetically pumped sheath-flow CE-MS nanospray interface.^26^ The cIEF separation was carried out using an 80 cm long linear polyacrylamide (LPA)-coated capillary (50 *μ*m i.d./360 *μ*m o.d.).^27,28^ The LPA coating was made according to refs 27 and 28. One end of the separation capillary was etched using hydrofluoric acid to reduce its outer diameter to approximately 100 *μ*m.^29^ The interface featured a glass spray emitter with an orifice size of 30–35 *μ*m, filled with a sheath buffer composed of 0.2% (v/v) formic acid and 10% (v/v) methanol. The spray voltage was set to 2 kV, and the capillary outlet to emitter orifice distance was maintained at approximately 0.5 mm. The distance between the emitter orifice and the MS inlet was about 2 mm.

The automated cIEF-MS system was based on the “sandwich” injection approach.^30–32^ The injection sequence involved three steps: first, a 6 cm catholyte plug was injected at 10 psi for 8 s containing 0.3% NH_4_OH, followed by a 20 cm mixture of sample and ampholyte plug containing 0.6% ampholytes (3–10, 5–8, and 8–10.5, GE Healthcare), injected at 10 psi for 27 s. Approximately 600 ng of corona proteins (1.5 mg/mL, injection volume of 400 nL) were loaded into the capillary, and finally, a 50 cm anolyte plug was injected at 10 psi for 67 s containing 5% acetic acid. This combination provided efficient focusing and mobilization of the protein corona samples under a separation voltage of 30 kV.

The Orbitrap Exploris 480 mass spectrometer was used to analyze the proteoforms separated by cIEF in data-dependent acquisition (DDA) mode. Two approaches were used for data acquisition to detect both small and large (>30 kDa) proteoforms. For small proteoforms (<30 kDa), we employed a “high-resolution MS1 and high-resolution MS/MS” mode, i.e., “High−High” mode. The detailed parameters for the “High−High” mode include MS1 resolution 480,000 at *m*/*z* 200 with a single microscan across a *m*/*z* range of 700–3000. Maximum ion injection time was set to 50 ms for MS and 100 ms for MS/MS. Normalized AGC target 300%, Ions with an intensity of over 1E4 and charge states varying from 5 to 60 were isolated with a 2 *m*/*z* window, followed by fragmentation through higher-energy collision dissociation (HCD) at 25% normalized collision energy (NCE). Dynamic exclusion was enabled with a duration of 30 s and a mass tolerance of 10 ppm, and isotope exclusion was activated. The fragment ions were detected with a resolution of 120 000 at *m*/*z* 200 and normalized AGC 100%. For large proteoforms (>30 kDa), a “low-resolution MS1 and high-resolution MS/MS” mode, i.e., “Low−High” mode, was employed. MS1 resolution of 7,500 at *m*/*z* 200 was used. The microscan setting is 3. The other parameters are the same as the “High−High” mode.

### Data Analysis.

Data processing was conducted using the TopPIC software developed by Liu’s group to identify and quantify proteoforms in the “High−High” mode.^33^ For the “Low−High” mode, the UniDec software facilitated mass deconvolution,^34^ determining the average masses of larger proteoforms. The cIEF-MS/MS data analysis began with converting RAW files to the mzML format using MSconvert. The converted data was then processed using TopFD (version 1.7.0) software^35^ to convert isotope clusters into monoisotopic masses and identifiable proteoform features, with the results stored in msalign and text files. The deconvoluted mass spectra and proteoform features were then searched against a home-built protein database of approximately 1,000 sequences using TopPIC software (version 1.7.0), which included proteins previously identified in bottom-up proteomics (BUP) data. TopPIC was configured to accommodate a single unexpected mass shift per proteoform with a maximum shift of 500 Da and maintained a mass error tolerance of 50 ppm for both precursor and fragment ions. A target-decoy approach was used to estimate and control the false discovery rate (FDR), setting it at 1% at the proteoform-spectrum match (PrSM) level and 5% at the proteoform level. Finally, the identified proteoforms were quantified using TopDiff software to enable label-free quantification across technical replicates. The quantification aggregated the intensities of each proteoform’s peaks across all scans and charge states.^36^ The raw mass spectrometry data files were processed using an Xcalibur Qual Browser (Thermo Fisher Scientific) to extract proteoform intensity values and migration time information. Base peak chromatograms and extracted ion chromatograms were generated to visualize the separation profiles. The electropherograms are graphically refined using Adobe Illustrator for figure preparation.

## RESULTS AND DISCUSSION

**Figure 1.**
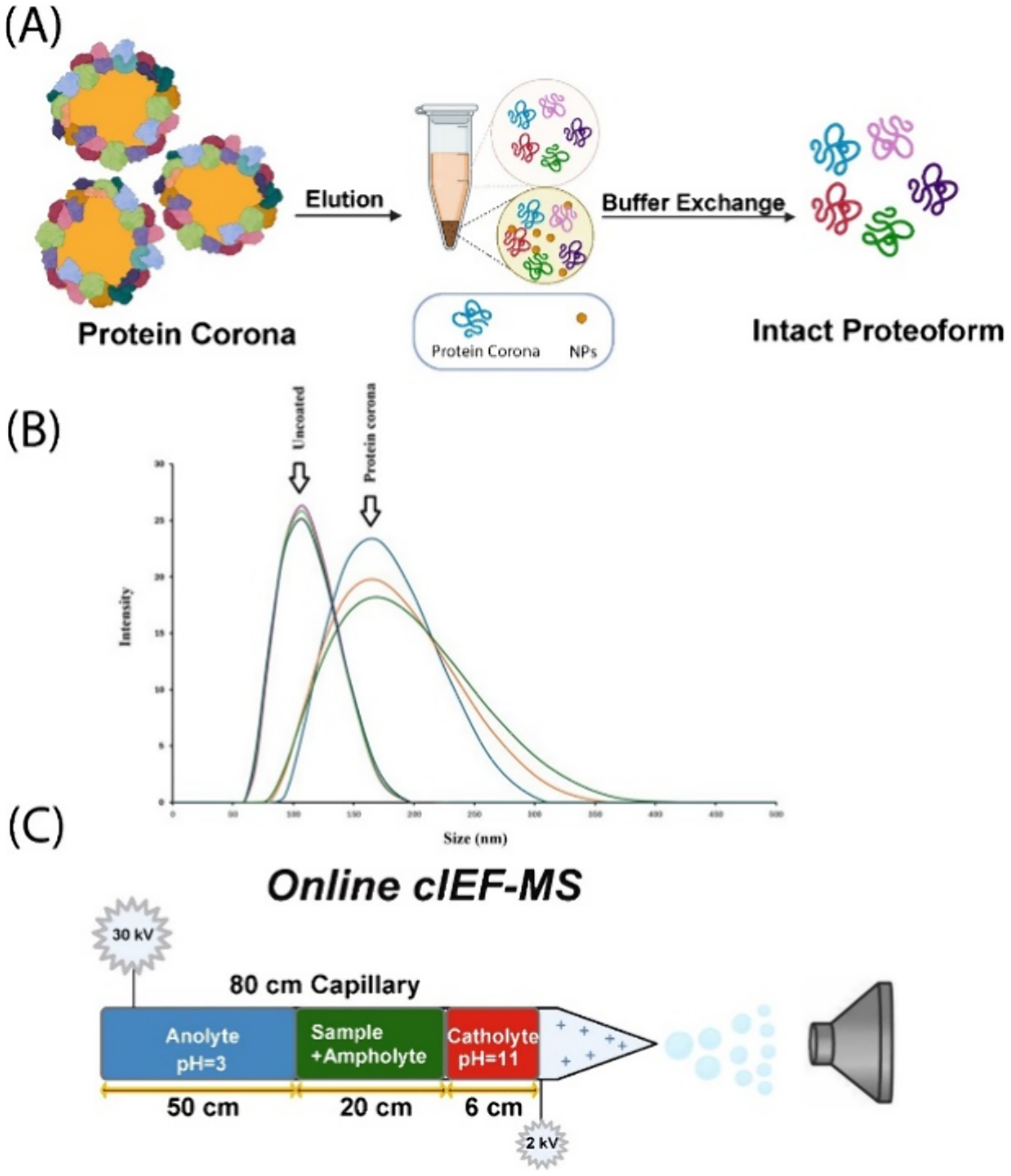
Schematic workflow of TDP of protein coronas using the advanced cIEF-MS/MS technique. (A) Brief workflow of preparing the protein corona sample for TDP after incubating the PSNPs with a human plasma sample to form the protein corona on the surface of PSNPs. (B) DLS analysis of bare PSNPs (uncoated) and protein corona coated NPs (protein corona). (C) Schematic design of the high-throughput cIEF-MS/MS for protein corona analysis.

We developed a high-throughput automated cIEF-MS/MS technique that took 30 min or less per run and applied it to the TDP of NP protein coronas (Figure 1). The protein corona sample was prepared according to our previous procedure.^29^ Briefly, proteoforms in the protein corona of PSNPs were eluted using a 0.4% SDS buffer and cleaned up by buffer exchange, followed by cIEF-MS/MS, Figure 1A. DLS analysis revealed protein corona formation at the surface of PSNPs, evidenced by increasing the size of corona-coated NPs, Figure 1B. The advanced cIEF-MS/MS technique for high-throughput TDP analysis of protein coronas was carried out by employing a short separation capillary for cIEF-MS with a commercial CE system, Figure 1C. An 80 cm long LPA-coated capillary was used, and the effective capillary length for cIEF separation was shorter than 30 cm because we used a “sandwich” injection approach, injecting a plug of catholyte (0.3% NH_4_OH, pH ~ 11), a plug of the sample with ampholyte in 100 mM NH_4_HCO_3_, and a long plug of anolyte (5% acetic acid, pH 2.4). We determined the optimal conditions as a 6 cm plug of catholyte, a 20 cm sample plug containing 0.6% ampholytes (pI 3–10, 5–8, and 8–10.5 with ratios 1:1:1), and a 50 cm plug of anolyte using a standard protein mixture (data not shown). Because of the short effective capillary length for cIEF (≤30 cm), the analysis could be carried out in a high-throughput fashion. Also, because the total capillary length was 80 cm, a regular commercial CE system could be used, allowing the technique to be easily adopted by other researchers.

### Reproducibility of High-Throughput cIEF-MS/MS-Based TDP for Protein Corona.

**Figure 2.**
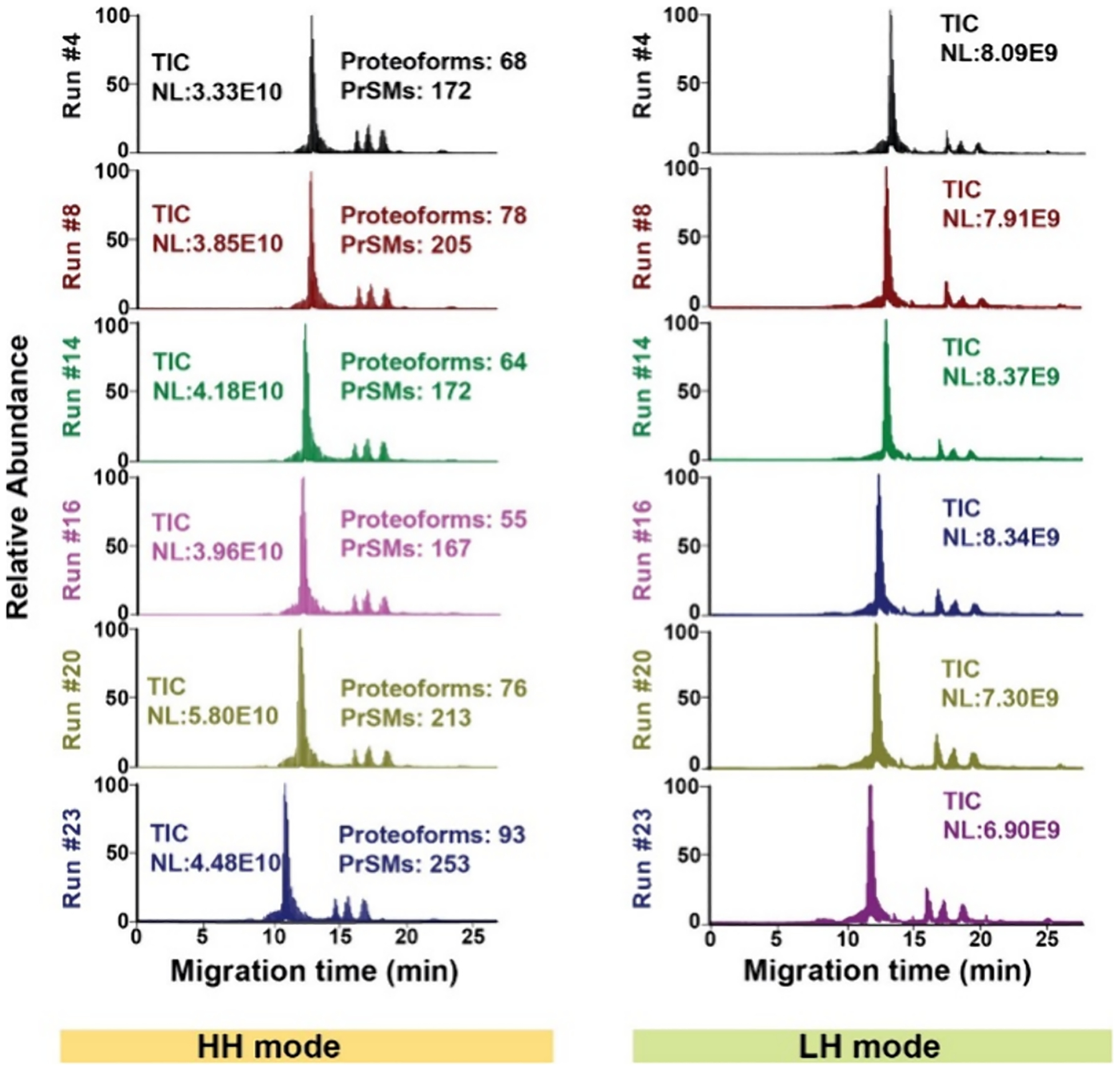
Total ion current (TIC) electropherograms of protein corona proteoforms as determined by CIEF-MS/MS in “High−High (HH)” and “Low−High (LH)” modes. Six selected electropherograms from runs #4, #8, #14, #16, #20, and #23 in HH and LH modes are shown.

We analyzed the protein corona sample of PSNPs using the optimal high-throughput cIEF-MS/MS technique for 50 runs, Figure 2. The 50 runs were performed on the same corona sample to evaluate the technical reproducibility of our cIEF-MS/MS method. Each cIEF-MS run took less than 30 min, producing a 2- to 6-fold improvement in analysis throughput compared to the previous cIEF-MS/MS-based TDP studies of complex samples.^24,26,30,37,38^ We performed 25 runs in the “High−High” mode and 25 runs in the “Low−High” mode to evaluate the technique for both small and large proteoform measurements.

**Figure 3.**
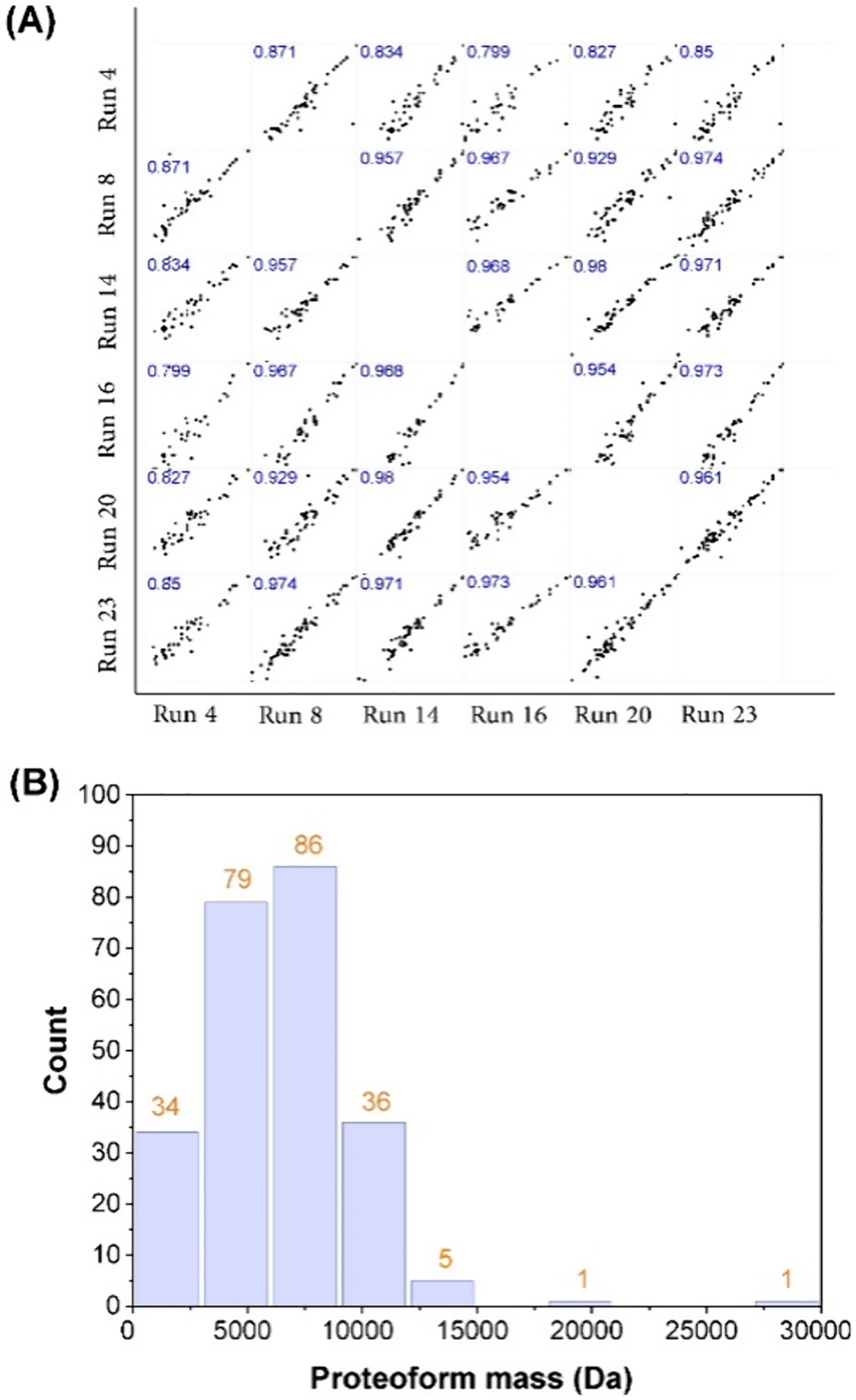
(A) Intensity correlations of overlapped proteoforms between any two cIEF-MS/MS runs. Six runs were randomly selected for this analysis. Proteoform intensities were log 2 transformed for the plot, and Pearson’s correlation coefficient (*r*) values were labeled. (B) Mass distribution of the identified proteoforms from 25 cIEF-MS/MS runs (High−High).

The cIEF-MS/MS technique produced reproducible separation, detection, and identification of proteoforms. The electropherograms in Figure 2A,B show consistent separation profiles of proteoforms in both “High−High” and “Low−High” modes. In the “High−High” mode, a normalized level (NL) of 4.0 ± 0.8 E10 (*n* = 25) was obtained for the total ion current (TIC) electropherograms, corresponding to a relative standard deviation (RSD) of 20%. In the “Low−High” mode, the NL was 8.2 ± 0.8E09, corresponding to an RSD of about 10%. The numbers of proteoform and proteoform-spectrum match (PrSM) identifications are also consistent across the “High−High” runs, with 71 ± 10 (*n* = 25) proteoforms and 196 ± 30 (*n* = 25) PrSMs. Importantly, the new cIEF-MS/MS technique produced a similar number of proteoform identifications to our previous cIEF-MS/MS technique for the same protein corona sample^24^ (~70 vs ~60) but with a 2-fold improvement in analysis throughput (30 vs 60 min). In the “Low−High” runs, three large proteins (28, 51, and 66 kDa) with multiple proteoforms per protein were consistently detected, Figure S1 in Supporting Information. Those three proteins correspond to human serum albumin (HSA, 66 kDa), apolipoprotein A-I (APOA1, 28 kDa), and an unknown protein (51 kDa). The data agreed reasonably with our previous results.^24,25^ We randomly selected seven proteoforms from seven genes and determined their migration times across the 25 “High−High” runs from the database search results to further evaluate the separation reproducibility, Table 1. The RSDs of the migration time of those proteoforms are less than 4% across 25 cIEF-MS/MS runs, indicating excellent reproducibility of the technique for proteoform separation. To validate the consistency of the high-throughput cIEF-MS/MS methodology for protein corona analysis regarding proteoform intensity, we randomly chose six cIEF-MS/MS runs (“High−High”) and plotted the intensity of overlapped proteoforms between any two runs, Figure 3A. The proteoform intensity showed strong linear correlations between runs, evidenced by the high Pearson’s correlation coefficient (*r*) of 0.92 ± 0.06, underscoring the quantitative reproducibility of the TDP technique for protein corona analysis. Figure 3B shows the mass distribution of identified proteoforms from all of the “High−High” runs. The mass of identified proteoforms ranged from ~2 kDa to ~30 kDa, and the majority of them were ~10 kDa or smaller. The identified proteoforms from each “High−High” run are listed in the Supporting Information xlsx file. We must point out that if we include the large proteoforms detected in “Low−High” mode, the mass range of identified proteoforms will be extended to 2–66 kDa.

### Protein Biomarkers Identified by cIEF-MS/MS Analysis of Protein Corona.

**Figure 4.**
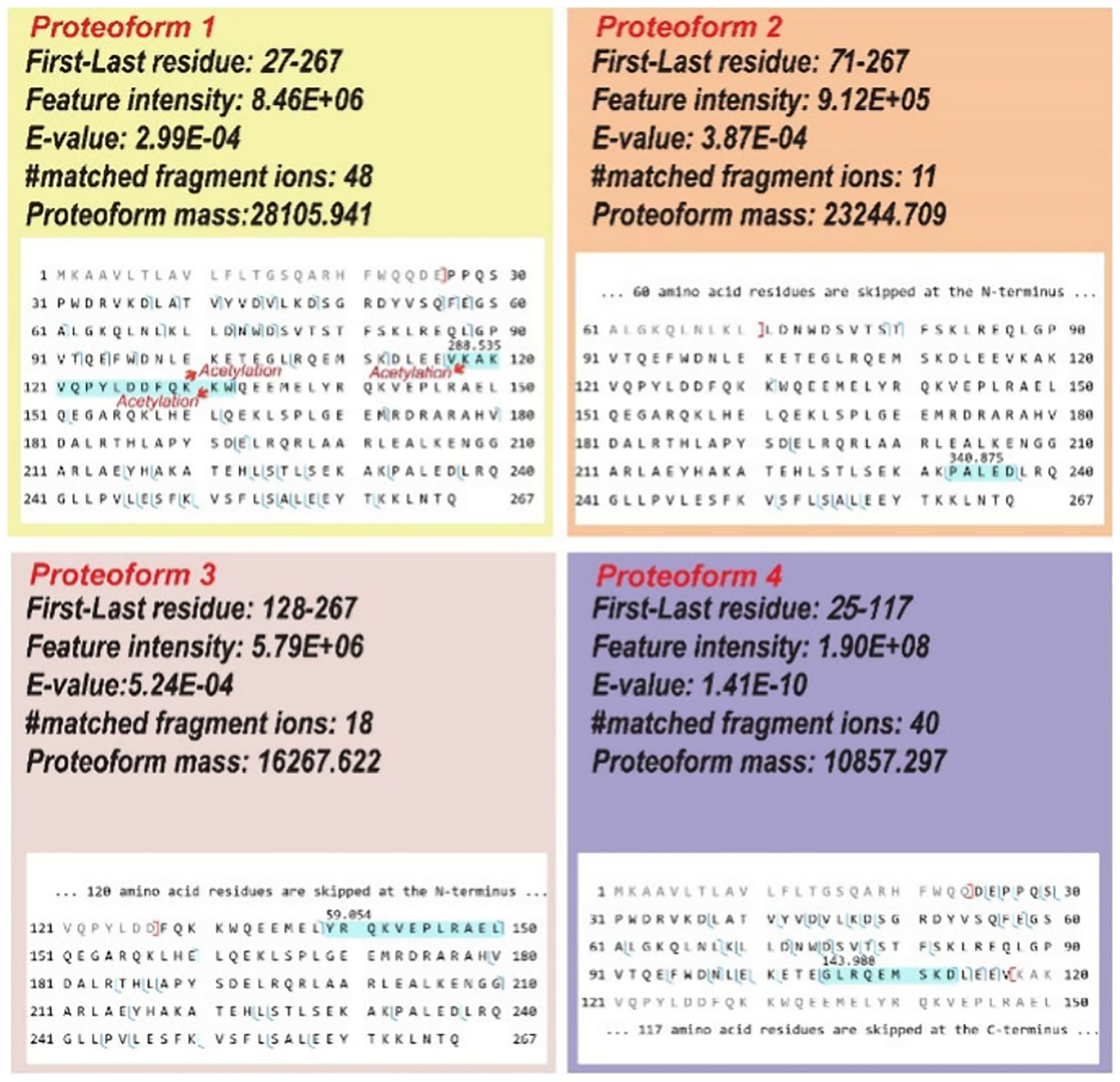
Sequences and fragmentation patterns of four distinct proteoforms of apolipoprotein A-I (APOA1) identified using cIEF-MS/MS-based TDP in “High−High” mode.

Our TDP analysis of the protein corona identified 53 genes, and the number of detected proteoforms per gene ranged from 1 to 102, Table 2. 33 out of the 53 genes are biomarkers, and they span various protein families and functional classes, including but not limited to apolipoproteins, complement proteins, immunoglobulins, and cytoskeletal proteins. Many of these proteins are associated with diverse diseases and pathological states, underscoring their potential utility as diagnostic or prognostic biomarkers.^39–43^ Particularly noteworthy is the prominence of the apolipoprotein family within the data set, which includes APOA1, APOA2, APOA4, APOB, APOC2, APOC3, APOE, and APOF. These proteins play critical roles in lipid metabolism and are strongly linked to cardiometabolic disorders such as dyslipidemia, metabolic syndrome, atherosclerosis, and cardiovascular diseases.^44–46^ This connection provides a significant opportunity for further research into their pathobiological mechanisms and applications in clinical diagnostics.^47–49^ Multiple proteoforms were identified for most of the apolipoprotein family members. For example, we identified 102 proteoforms of the *APOA1* gene. Nearly 90% are N-terminally truncated, and only several proteoforms have C-terminal truncations. About 85% of proteoforms carry mass shifts due to sequence variations or modifications. Four *APOA1* proteoforms are shown in Figure 4. Those proteoforms carry variations due to signal peptide cleavages, truncations, and PTMs. Proteoform 1 has an N-terminal cleavage of the first 26 amino acid residues, most likely corresponding to signal peptide cleavage. Proteoform 1 also contains a mass shift of +288.535 Da in the highlighted region. Based on the PTM information in the dbPTM database (https://awi.cuhk.edu.cn/dbPTM/),^50^ three lysine residues in the highlighted region can be acetylated, corresponding to a +126 Da mass shift. The +288.535 Da value may correspond to the combination of acetylation and other PTMs. For proteoform 2, the first 70 amino acid residues were truncated, and it carries a mass shift of +340.875 Da. Proteoform 3 shows a truncation of the first 127 amino acid residues at the N-terminus. This proteoform also contains an unknown mass shift of +59.054 Da in the highlighted region. The exact nature of this modification is required for further investigation. Proteoform 4 exhibits an N-terminal removal of the first 24 amino acid residues due to signal peptide cleavage and a C-terminal truncation. This proteoform also contains an unknown mass shift of +143.988 Da in the highlighted region. Additionally, the data set identifies biomarkers pertinent to inflammatory and autoimmune diseases, including complement proteins (C3, C9),^51–53^ serum amyloid A proteins (SAA1), and serpins (SERPINA1, SERPINC1).^51,54^ The catalog also highlights biomarkers associated with neurodegenerative conditions such as Alzheimer’s disease (APOE, CLU)^55,56^ and Parkinson’s disease (ABCB9),^57^ as well as proteins involved in cancer progression and metastasis, such as ACTB, KRT1, and RAB15.^58–61^ These findings demonstrate that TDP studies of protein coronas from large cohorts of plasma samples with various diseases using our high-throughput cIEF-MS/MS technique could discover novel proteoform biomarkers of diseases, facilitating disease early diagnosis and drug development.

## CONCLUSIONS

A high-throughput cIEF-MS/MS technique was developed to profile the proteoform composition of protein coronas with excellent qualitative and quantitative reproducibility across 50 runs. The number of proteoform identifications per cIEF-MS/MS run for protein coronas is comparable with our previous cIEF-MS/MS data^24,25^ but with a 2-fold improvement in analysis throughput (30 min per run vs 60 min per run). Our advanced cIEF-MS/MS technique is ready for quantitative TDP analysis of large cohorts of human plasma samples using the protein corona approach to discover novel proteoform biomarkers of various diseases.

We also must note that the number of proteoform identifications from our high-throughput cIEF-MS/MS is lower than that produced by CZE-MS/MS^23^ for the same protein corona sample (~70 vs ~100), although cIEF-MS/MS has a 2-fold higher analysis throughput than CZE-MS/MS (30 vs 60 min per run). The reason is mainly due to the significant ionization suppression of proteoforms from high-concentration ampholytes in cIEF-MS/MS. We expect that integrating FAIMS (high field asymmetric waveform ion mobility spectrometry)^62^ with our fast cIEF-MS/MS may be useful to advance the technique further toward better proteoform coverage of the protein corona samples because FAIMS can potentially separate ampholytes from proteoforms in the gas phase due to their substantial difference in size.

## Supplementary Material

Supporting Information I

Supporting Information II

## Figures and Tables

**Table 1.** Summary of Migration Times of Seven Selected Proteoforms from Seven Proteins across 25 “High−High” Runs

protein	migration time (mean, s)	Standard deviation (SD, s)	relative standard deviation (RSD, %)
Apolipoprotein E	899.81	19.8	2.2
Apolipoprotein A-II	881.8	22.0	2.5
Apolipoprotein C-II	1008.6	34.4	3.4
Apolipoprotein A-I	949.8	22.2	2.3
Fibrinogen *α* chain	806.0	19.3	2.4
Apolipoprotein C-III	1589.3	62.4	3.9
Clusterin	824.1	24.4	3.0

**Table 2.** Summary of the Identified Genes and Corresponding Number of Proteoforms from the cIEF-MS/MS-Based TDP Analysis of Nanoparticle Protein Coronas

gene name	no. of proteoforms	protein description	biomarker
ACTB	1	Actin beta	cancer, neurological disorders, and cardiovascular diseases.
ACTG2 (F8WCH0)	1	Actin gamma 2, smooth muscle	
ALB	1	Albumin	cardiovascular diseases, liver function, inflammation, and malnutrition.
SERPINA1 (A1AT)	9	Alpha-1-antitrypsin	lung diseases, such as chronic obstructive pulmonary disease (COPD) and emphysema.
SERPINA1 (G3V387)	1	Alpha-1-antitrypsin (fragment)	
SERPINA2 (A1ATR)	1	Alpha-1-antitrypsin-related protein	
APOA1	102	Apolipoprotein A-I	cardiovascular diseases, diabetes, and certain types of cancer
APOA2	24	Apolipoprotein A-II	cardiometabolic conditions such as dyslipidemia, metabolic syndrome, and atherosclerosis.
APOA2 (V9GYM3)	7	Apolipoprotein A-II	
APOA2 (V9GYS1)	20	Apolipoprotein A-II	
APOA2 (V9GYC1)	1	Apolipoprotein A-II (fragment)	
APOA4	8	Apolipoprotein A-IV	cardiometabolic conditions such as dyslipidemia, metabolic syndrome, and atherosclerosis.
APOB	14	Apolipoprotein B-100	cardiovascular diseases, such as atherosclerosis and heart disease.
APOC2	30	Apolipoprotein C-II	cardiometabolic conditions such as dyslipidemia, metabolic syndrome, and atherosclerosis.
APOC2 (V9GYJ8)	14	Apolipoprotein C-II	
APOC2 (Q6P163)	2	Apolipoprotein C-II	
APOC3	43	Apolipoprotein C-III	cardiovascular diseases and metabolic disorders, such as hypertriglyceridemia
APOC3 (BOYIW2)	4	Apolipoprotein C-III	
APOC3 (C9J2Q0)	3	Apolipoprotein C-III (fragment)	
APOE	14	Apolipoprotein E	Alzheimer’s disease and other neurodegenerative disorders.
APOF	1	Apolipoprotein F	cardiometabolic conditions such as dyslipidemia, metabolic syndrome, and atherosclerosis.
APOL1	1	Apolipoprotein L1	kidney disease, especially in high-risk populations.
SERPINC1 (ANT3)	1	Apolipoprotein L1	
ABCB9	1	ATP binding cassette subfamily B member 9 (fragment)	Parkinson’s disease and Alzheimer’s disease.
CPN2	1	Carboxypeptidase N subunit 2	sepsis and acute pancreatitis.
CLU	3	Clusterin	neurodegenerative disorders, cancer, and kidney disease.
C1R	1	Complement C1r (fragment)	may have potential for the systemic lupus erythematosus and rheumatoid arthritis
C1RL	1	Complement C1r subcomponent like (fragment)	may have potential for inflammatory and autoimmune disorders, such as systemic lupus erythematosus and rheumatoid arthritis
C3	15	Complement C3	inflammatory and autoimmune diseases, such as rheumatoid arthritis and systemic lupus erythematosus.
C9	1	Complement C9	inflammatory and autoimmune diseases, such as systemic lupus erythematosus and rheumatoid arthritis.
FETUB	1	Fetuin B (Fragment)	may have potential for metabolic disorders, such as chronic kidney disease and nonalcoholic fatty liver disease (NAFLD)
FGA	7	Fibrinogen alpha chain	cardiovascular diseases, thrombosis, and inflammation.
FGB	7	Fibrinogen beta chain	
FGG	5	Fibrinogen gamma chain	
GSN	1	Gelsolin	Alzheimer’s disease, amyloidosis, and critical illness
PLG	1	HCG2029799, isoform CRA d	
IGLV2–8	1	Immunoglobulin lambda variable 2–8	
IGLL5	1	Immunoglobulin lambda-like polypeptide 5	hematological cancers.
ITIH1	4	Interalpha-trypsin inhibitor heavy chain	cancer, liver disease, and inflammatory conditions
ITIH4	3	Interalpha-trypsin inhibitor heavy chain H4	
KRT1	1	Keratin, type II cytoskeletal 1	epithelial cancers, such as lung, breast, and prostate cancer.
KRT71	1	Keratin, type II cytoskeletal 71	
KRT74	1	Keratin, type II cytoskeletal 74	
DEFA1	1	Neutrophil defensin 1	infection severity such as acute respiratory distress syndrome (ARDS) and sepsis.
RAB15	1	RAB15, member RAS oncogene family	certain cancers, such as lung, pancreatic, and colorectal cancer.
RAP1B	1	RAP1B, member of RAS oncogene family (fragment)	may have potential for certain cancers, such as lung, pancreatic, and colorectal cancer.
SELENOP	1	Selenoprotein P (fragment)	may have potential for various conditions, including oxidative stress, inflammation, and metabolic disorders.
SAA1 (E9PQD6)	6	Serum amyloid A protein	May have potential for various inflammatory conditions and neoplastic disorders, such as rheumatoid arthritis, Crohn’s disease, and certain types of cancer.
SAA1	14	Serum amyloid A-1 protein	various inflammatory conditions, such as rheumatoid arthritis Crohn’s disease, and certain types of cancer.
SHBG	1	Sex hormone binding globulin	
TTR	7	Transthyretin	amyloidosis, a group of diseases characterized by the accumulation of abnormal proteins in tissues.
TTR (A0A087WT59)	2	Transthyretin	
TUBA1C	1	Tubulin alpha chain	cancer, neurodegenerative disorders, and certain types of ciliopathies.

## Data Availability

The MS RAW files about TDP measurement were deposited to the ProteomeXchange Consortium via PRIDE, with the data set identifier PXD052764.^63^

## References

[1] Pelaz B; Alexiou C; Alvarez-Puebla RA; Alves F; Andrews AM; Ashraf S; Balogh LP; Ballerini L; Bestetti A; Brendel C; Bosi S; Carril M; Chan WCW; Chen C; Chen X; Chen X; Cheng Z; Cui D; Du J; Dullin C; Escudero A; Feliu N; Gao M; George M; Gogotsi Y; Grünweller A; Gu Z; Halas NJ; Hampp N; Hartmann RK; Hersam MC; Hunziker P; Jian J; Jiang X; Jungebluth P; Kadhiresan P; Kataoka K; Khademhosseini A; Kopeček J; Kotov NA; Krug HF; Lee DS; Lehr CM; Leong KW; Liang XJ; Lim ML; Liz-Marzán LM; Ma X; Macchiarini P; Meng H; Möhwald H; Mulvaney P; Nel AE; Nie S; Nordlander P; Okano T; Oliveira J; Park TH; Penner RM; Prato M; Puntes V; Rotello VM; Samarakoon A; Schaak RE; Shen Y; Sjöqvist S; Skirtach AG; Soliman MG; Stevens MM; Sung HW; Tang BZ; Tietze R; Udugama BN; VanEpps JS; Weil T; Weiss PS; Willner I; Wu Y; Yang L; Yue Z; Zhang Q; Zhang Q; Zhang XE; Zhao Y; Zhou X; Parak WJ Diverse Applications of Nanomedicine. ACS Nano 2017, 11 (3), 2313–2381. 28290206 10.1021/acsnano.6b06040 PMC5371978

[2] Faria M; Björnmalm M; Thurecht KJ; Kent SJ; Parton RG; Kavallaris M; Johnston APR; Gooding JJ; Corrie SR; Boyd BJ; Thordarson P; Whittaker AK; Stevens MM; Prestidge CA; Porter CJH; Parak WJ; Davis TP; Crampin EJ; Caruso F Minimum Information Reporting in Bio-Nano Experimental Literature. Nat. Nanotechnol 2018, 13 (9), 777–785. 30190620 10.1038/s41565-018-0246-4 PMC6150419

[3] Mitchell MJ; Billingsley MM; Haley RM; Wechsler ME; Peppas NA; Langer R Engineering Precision Nanoparticles for Drug Delivery. Nat. Rev. Drug Discovery 2021, 20, 101–124. 33277608 10.1038/s41573-020-0090-8 PMC7717100

[4] Sadeghi A; PourEskandar S; Askari E; Akbari M Polymeric Nanoparticles and Nanogels: How Do They Interact with Proteins? Gels 2023, 9, 632. 37623087 10.3390/gels9080632 PMC10453451

[5] Hajipour MJ; Laurent S; Aghaie A; Rezaee F; Mahmoudi M Personalized Protein Coronas: A “Key” Factor at the Nanobiointerface. Biomater Sci. 2014, 2 (9), 1210–1221. 32481892 10.1039/c4bm00131a

[6] Patra JK; Das G; Fraceto LF; Campos EVR; Rodriguez-Torres MDP; Acosta-Torres LS; Diaz-Torres LA; Grillo R; Swamy MK; Sharma S; Habtemariam S; Shin HS Nano Based Drug Delivery Systems: Recent Developments and Future Prospects. J. Nanobiotechnol 2018, 16, 71. 10.1186/s12951-018-0392-8 PMC6145203 30231877

[7] Bertrand N; Grenier P; Mahmoudi M; Lima EM; Appel EA; Dormont F; Lim JM; Karnik R; Langer R; Farokhzad OC Mechanistic Understanding of in Vivo Protein Corona Formation on Polymeric Nanoparticles and Impact on Pharmacokinetics. Nat. Commun 2017, 8 (1), 777. 28974673 10.1038/s41467-017-00600-w PMC5626760

[8] Walczyk D; Bombelli FB; Monopoli MP; Lynch I; Dawson KA What the Cell “Sees” in Bionanoscience. J. Am. Chem. Soc 2010, 132 (16), 5761–5768. 20356039 10.1021/ja910675v

[9] Corbo C; Li AA; Poustchi H; Lee GY; Stacks S; Molinaro R; Ma P; Platt T; Behzadi S; Langer R; Farias V; Farokhzad OC Analysis of the Human Plasma Proteome Using Multi-Nanoparticle Protein Corona for Detection of Alzheimer’s Disease. Adv. Healthcare Mater 2021, 10 (2), 2000948. 10.1002/adhm.202000948 33169521

[10] Mahmoudi M; Landry MP; Moore A; Coreas R The Protein Corona from Nanomedicine to Environmental Science. Nat. Rev. Mater 2023, 8, 422–438. 10.1038/s41578-023-00552-2 PMC10037407 37361608

[11] Ren J; Cai R; Wang J; Daniyal M; Baimanov D; Liu Y; Yin D; Liu Y; Miao Q; Zhao Y; Chen C Precision Nanomedicine Development Based on Specific Opsonization of Human Cancer Patient-Personalized Protein Coronas. Nano Lett. 2019, 19 (7), 4692–4701. 31244235 10.1021/acs.nanolett.9b01774

[12] Ashkarran AA; Gharibi H; Voke E; Landry MP; Saei AA; Mahmoudi M Measurements of Heterogeneity in Proteomics Analysis of the Nanoparticle Protein Corona across Core Facilities. Nat. Commun 2022, 13 (1), 6610. 36329043 10.1038/s41467-022-34438-8 PMC9633814

[13] Jiang Y; Rex DAB; Schuster D; Neely BA; Rosano GL; Volkmar N; Momenzadeh A; Peters-Clarke TM; Egbert SB; Kreimer S; Doud EH; Crook OM; Yadav AK; Vanuopadath M; Hegeman AD; Mayta ML; Duboff AG; Riley NM; Moritz RL; Meyer JG Comprehensive Overview of Bottom-Up Proteomics Using Mass Spectrometry. ACS Measur. Sci. Au 2024, 4, 338–417. 10.1021/acsmeasuresciau.3c00068 PMC11348894 39193565

[14] Miller RM; Smith LM Overview and Considerations in Bottom-up Proteomics. Analyst 2023, 148, 475–486. 36383138 10.1039/d2an01246d PMC9898146

[15] Smith LM; Agar JN; Chamot-Rooke J; Danis PO; Ge Y; Loo JA; Paša-Tolić L; Tsybin YO; Kelleher NL; Consortium for Top-Down Proteomics. Human Proteoform Project: Defining the human proteome. Sci. Adv 2021, 7, eabk0734. 34767442 10.1126/sciadv.abk0734 PMC8589312

[16] Smith LM; Kelleher NL Proteoforms as the next Proteomics Currency. Science 2018, 359 (6380), 1106–1107. 29590032 10.1126/science.aat1884 PMC5944612

[17] Saei AA; Sun L; Mahmoudi M The Role of Protein Corona in Advancing Plasma Proteomics. Proteomics 2025, 25, e2400028 DOI: 10.1002/pmic.202400028. 39221533 PMC11735278

[18] Blume JE; Manning WC; Troiano G; Hornburg D; Figa M; Hesterberg L; Platt TL; Zhao X; Cuaresma RA; Everley PA; Ko M; Liou H; Mahoney M; Ferdosi S; Elgierari EM; Stolarczyk C; Tangeysh B; Xia H; Benz R; Siddiqui A; Carr SA; Ma P; Langer R; Farias V; Farokhzad OC Rapid, Deep and Precise Profiling of the Plasma Proteome with Multi-Nanoparticle Protein Corona. Nat. Commun 2020, 11 (1), 3662. 32699280 10.1038/s41467-020-17033-7 PMC7376165

[19] Chen B; Brown KA; Lin Z; Ge Y Top-Down Proteomics: Ready for Prime Time? Anal. Chem 2018, 90, 110–127. 29161012 10.1021/acs.analchem.7b04747 PMC6138622

[20] Tran JC; Zamdborg L; Ahlf DR; Lee JE; Catherman AD; Durbin KR; Tipton JD; Vellaichamy A; Kellie JF; Li M; Wu C; Sweet SMM; Early BP; Siuti N; Leduc RD; Compton PD; Thomas PM; Kelleher NL Mapping Intact Protein Isoforms in Discovery Mode Using Top-down Proteomics. Nature 2011, 480 (7376), 254–258. 22037311 10.1038/nature10575 PMC3237778

[21] Donnelly DP; Rawlins CM; DeHart CJ; Fornelli L; Schachner LF; Lin Z; Lippens JL; Aluri KC; Sarin R; Chen B; Lantz C; Jung W; Johnson KR; Koller A; Wolff JJ; Campuzano IDG; Auclair JR; Ivanov AR; Whitelegge JP; Paša-Tolić L; Chamot-Rooke J; Danis PO; Smith LM; Tsybin YO; Loo JA; Ge Y; Kelleher NL; Agar JN Best Practices and Benchmarks for Intact Protein Analysis for Top-down Mass Spectrometry. Nat. Methods 2019, 16 (7), 587–594. 31249407 10.1038/s41592-019-0457-0 PMC6719561

[22] Catherman AD; Durbin KR; Ahlf DR; Early BP; Fellers RT; Tran JC; Thomas PM; Kelleher NL Large-Scale Top-down Proteomics of the Human Proteome: Membrane Proteins, Mitochondria, and Senescence. Mol. Cell. Proteomics 2013, 12 (12), 3465–3473. 24023390 10.1074/mcp.M113.030114 PMC3861700

[23] Sadeghi SA; Ashkarran AA; Wang Q; Zhu G; Mahmoudi M; Sun L Mass Spectrometry-Based Top-Down Proteomics in Nanomedicine: Proteoform-Specific Measurement of Protein Corona. ACS Nano 2024, 18 (38), 26024–26036. 39276099 10.1021/acsnano.4c04675 PMC11440641

[24] Zhu G; Sadeghi SA; Mahmoudi M; Sun L Deciphering Nanoparticle Protein Coronas by Capillary Isoelectric Focusing-Mass Spectrometry-Based Top-down Proteomics. Chem. Commun 2024, 60 (81), 11528–11531. 10.1039/d4cc02666g PMC11418007 39310940

[25] Sadeghi SA; Ashkarran AA; Mahmoudi M; Sun L Mass spectrometry-based top-down proteomics in nanomedicine: proteoform-specific measurement of protein corona. bioRxiv 2024, DOI: 10.1101/2024.03.22.586273. PMC11440641 39276099

[26] Xu T; Shen X; Yang Z; Chen D; Lubeckyj RA; McCool EN; Sun L Automated Capillary Isoelectric Focusing-Tandem Mass Spectrometry for Qualitative and Quantitative Top-Down Proteomics. Anal. Chem 2020, 92 (24), 15890–15898. 33263984 10.1021/acs.analchem.0c03266 PMC8564864

[27] Chen D; Shen X; Sun L Capillary Zone Electrophoresis-Mass Spectrometry with Microliter-Scale Loading Capacity, 140 min Separation Window and High Peak Capacity for Bottom-up Proteomics. Analyst 2017, 142 (12), 2118−2127. 28513658 10.1039/c7an00509a

[28] Zhu G; Sun L; Dovichi NJ Dynamic PH Junction Preconcentration in Capillary Electrophoresis- Electrospray Ionization-Mass Spectrometry for Proteomics Analysis. Analyst 2016, 141, 5216–5220. 27460877 10.1039/c6an01140c PMC5007160

[29] Sun L; Zhu G; Zhao Y; Yan X; Mou S; Dovichi NJ Ultrasensitive and Fast Bottom-up Analysis of Femtogram Amounts of Complex Proteome Digests. Angewandte Chemie - International Edition 2013, 52 (51), 13661–13664. 24173663 10.1002/anie.201308139 PMC3904452

[30] Xu T; Han L; George Thompson AM; Sun L An Improved Capillary Isoelectric Focusing-Mass Spectrometry Method for High-Resolution Characterization of Monoclonal Antibody Charge Variants. Analytical Methods 2022, 14 (4), 383–393. 34939625 10.1039/d1ay01556g

[31] Zhu G; Sun L; Dovichi NJ Simplified Capillary Isoelectric Focusing with Chemical Mobilization for Intact Protein Analysis. J. Sep Sci 2017, 40 (4), 948–953. 27935257 10.1002/jssc.201601051

[32] Xu T; Sun L A Mini Review on Capillary Isoelectric Focusing-Mass Spectrometry for Top-Down Proteomics. Front. Chem 2021, 9, 651757. 33898392 10.3389/fchem.2021.651757 PMC8063032

[33] Kou Q; Xun L; Liu X TopPIC: A Software Tool for Top-down Mass Spectrometry-Based Proteoform Identification and Characterization. Bioinformatics 2016, 32 (22), 3495–3497. 27423895 10.1093/bioinformatics/btw398 PMC5181555

[34] Marty MT; Baldwin AJ; Marklund EG; Hochberg GKA; Benesch JLP; Robinson CV Bayesian Deconvolution of Mass and Ion Mobility Spectra: From Binary Interactions to Polydisperse Ensembles. Anal. Chem 2015, 87 (8), 4370–4376. 25799115 10.1021/acs.analchem.5b00140 PMC4594776

[35] Basharat AR; Zang Y; Sun L; Liu X TopFD: A Proteoform Feature Detection Tool for Top-Down Proteomics. Anal. Chem 2023, 95 (21), 8189–8196. 37196155 10.1021/acs.analchem.2c05244 PMC10233584

[36] Lubeckyj RA; Basharat AR; Shen X; Liu X; Sun L Large-Scale Qualitative and Quantitative Top-Down Proteomics Using Capillary Zone Electrophoresis-Electrospray Ionization-Tandem Mass Spectrometry with Nanograms of Proteome Samples. J. Am. Soc. Mass Spectrom 2019, 30 (8), 1435–1445. 30972727 10.1007/s13361-019-02167-w PMC6675661

[37] Fang F; Xu T; Hagar H-TC; Hovde S; Kuo M-H; Sun L A Pilot Study for Deciphering Post-Translational Modifications and Proteoforms of Tau Protein by Capillary Electrophoresis-Mass Spectrometry. J. Proteome Res 2024, 23, 5085. 39327902 10.1021/acs.jproteome.4c00587 PMC11536466

[38] Xu T; Han L; Sun L Automated Capillary Isoelectric Focusing-Mass Spectrometry with Ultrahigh Resolution for Characterizing Microheterogeneity and Isoelectric Points of Intact Protein Complexes. Anal. Chem 2022, 94 (27), 9674–9682. 35766479 10.1021/acs.analchem.2c00975

[39] Xu R; Shen J; Song Y; Lu J; Liu Y; Cao Y; Wang Z; Zhang J Exploration of the Application Potential of Serum Multi-Biomarker Model in Colorectal Cancer Screening. Sci. Rep 2024, 14 (1), 10127. 38698075 10.1038/s41598-024-60867-0 PMC11066011

[40] Pitkänen HH; Haapio M; Saarela M; Taskinen MR; Brinkman HJ; Lassila R Impact of Therapeutic Plasma Exchange on Intact Protein S, Apolipoproteins, and Thrombin Generation. Transfusion and Apheresis Science 2024, 63, 103918. 38555232 10.1016/j.transci.2024.103918

[41] Retracted: Exploration of Potential Biomarkers and Immune Landscape for Hepatoblastoma: Evidence from Machine Learning Algorithm. Evidence-Based Complementary and Alternative Medicine 2023, 2023, 9893765,. 37886423 10.1155/2023/9893765 PMC10599939

[42] Zhu M; Lan Z; Park J; Gong S; Wang Y; Guo F Regulation of CNS Pathology by Serpina3n/SERPINA3: The Knowns and the Puzzles. Neuropathol. Appl. Neurobiol 2024, 50, e12980. 10.1111/nan.12980 PMC11131959 38647003

[43] Neagu A-N; Whitham D; Buonanno E; Jenkins A; Alexa-Stratulat T; Tamba BI; Darie CC Proteomics and Its Applications in Breast Cancer. Am. J. Cancer Res 2021, 11, 4006. 34659875 PMC8493401

[44] Schreiner TG; Ignat BE; Grosu C; Costache AD; Leon MM; Mitu F Lipid-Derived Biomarkers as Therapeutic Targets for Chronic Coronary Syndrome and Ischemic Stroke: An Updated Narrative Review. Medicina 2024, 60, 561. 38674207 10.3390/medicina60040561 PMC11052465

[45] Wu B; Yang X; Chen F; Song Z; Ding X; Wang X Apolipoprotein E Is a Prognostic Factor for Pancreatic Cancer and Associates with Immune Infiltration. Cytokine 2024, 179, 156628. 38704962 10.1016/j.cyto.2024.156628

[46] Churchill RA; Gochanour BR; Scott CG; Vasile VC; Rodeheffer RJ; Meeusen JW; Jaffe AS Association of Cardiac Biomarkers with Long-Term Cardiovascular Events in a Community Cohort. Biomarkers 2024, 29, 161. 38666319 10.1080/1354750X.2024.2335245

[47] Han S; Zhang J; Sun Y; Liu L; Guo L; Zhao C; Zhang J; Qian Q; Cui B; Zhang Y The Plasma DIA-Based Quantitative Proteomics Reveals the Pathogenic Pathways and New Biomarkers in Cervical Cancer and High Grade Squamous Intraepithelial Lesion. J. Clin Med 2022, 11 (23), 7155. 36498728 10.3390/jcm11237155 PMC9736146

[48] Zhu Y; Zhang H; Jiang P; Xie C; Luo Y; Chen J Transcriptional and Epigenetic Alterations in the Progression of Non-Alcoholic Fatty Liver Disease and Biomarkers Helping to Diagnose Non-Alcoholic Steatohepatitis. Biomedicines 2023, 11 (3), 970. 36979950 10.3390/biomedicines11030970 PMC10046227

[49] Lv JH; Hou AJ; Zhang SH; Dong JJ; Kuang HX; Yang L; Jiang H WGCNA Combined with Machine Learning to Find Potential Biomarkers of Liver Cancer. Medicine 2023, 102 (50), No. e36536. 38115320 10.1097/MD.0000000000036536 PMC10727608

[50] Li Z; Li S; Luo M; Jhong JH; Li W; Yao L; Pang Y; Wang Z; Wang R; Ma R; Yu J; Huang Y; Zhu X; Cheng Q; Feng H; Zhang J; Wang C; Hsu JBK; Chang WC; Wei FX; Huang H Da; Lee, T. Y. DbPTM in 2022: An Updated Database for Exploring Regulatory Networks and Functional Associations of Protein Post-Translational Modifications. Nucleic Acids Res. 2022, 50 (D1), D471–D479. 34788852 10.1093/nar/gkab1017 PMC8728263

[51] Rodriguez-Muñoz A; Motahari-Rad H; Martin-Chaves L; Benitez-Porres J; Rodriguez-Capitan J; Gonzalez-Jimenez A; Insenser M; Tinahones FJ; Murri M 92A Systematic Review of Proteomics in Obesity: Unpacking the Molecular Puzzle. Curr. Obes Rep 2024, 13, 403. 38703299 10.1007/s13679-024-00561-4 PMC11306592

[52] Hu C; Zhao Z; Dong S; Guo Q; Zhou Y The Clinical Role of Combined Circulating Complement C1q and AIP for CAD with LDL-C Level below 1.8mmol/L. Lipids Health Dis 2024, 23 (1), 131. 38704561 10.1186/s12944-024-02127-8 PMC11070092

[53] Chrisman M; White-Lewis S; Lasiter S; Chesnut SR; Russell CL Equine-Assisted Service’s Effect on Cartilage and Skeletal Biomarkers for Adults and Older Adults with Arthritis: A Pilot Study. Complement Ther Med. 2024, 82, 103047. 38697487 10.1016/j.ctim.2024.103047

[54] Nady A; Reichheld SE; Sharpe S Structural Studies of a Serum Amyloid A Octamer That Is Primed to Scaffold Lipid Nanodiscs. Protein Sci. 2024, 33 (5), e4983. 38659173 10.1002/pro.4983 PMC11043621

[55] Sołkiewicz K; Kokot I; Kacperczyk M; Dymicka-Piekarska V; Dorf J; Kratz EM Serum Clusterin Concentration and Its Glycosylation Changes as Potential New Diagnostic Markers of SARS-CoV-2 Infection and Recovery Process. Int. J. Mol. Sci 2024, 25 (8), 4198. 38673784 10.3390/ijms25084198 PMC11049940

[56] Chen X-X; Zeng M-X; Cai D; Zhou H-H; Wang Y-J; Liu Z Correlation between APOE4 Gene and Gut Microbiota in Alzheimer’s Disease. Benef Microbes 2023, 14 (4), 349–360. 38661357 10.1163/18762891-20220116

[57] Hou L; Zhang X; Jiao Y; Li Y; Zhao Y; Guan Y; Liu Z ATP Binding Cassette Subfamily B Member 9 (ABCB9) Is a Prognostic Indicator of Overall Survival in Ovarian Cancer. Medicine 2019, 98 (19), e15698. 31083274 10.1097/MD.0000000000015698 PMC6531167

[58] Li J; Yan W; Yuan H; Ren F Theacrine Enhances Autophagy and Inhibits Inflammation via Regulating SIRT3/FOXO3a/Parkin Pathway. Int. J. Rheum. Dis 2024, 27 (2), e15085. 38402443 10.1111/1756-185X.15085

[59] Jiang X; Yang L; Gao Q; Liu Y; Feng X; Ye S; Yang Z The Role of RAB GTPases and Its Potential in Predicting Immunotherapy Response and Prognosis in Colorectal Cancer. Front. Genet 2022, 13, 828373. 35154286 10.3389/fgene.2022.828373 PMC8833848

[60] Zhao Q; Wu Y; Wu X; Liu M; Nan L Single-Cell Transcriptome Analysis Reveals Keratinocyte Subpopulations Contributing to Psoriasis in Corneum and Granular Layer. Skin Res. Technol 2024, 30 (2), e13572. 38279596 10.1111/srt.13572 PMC10818132

[61] Shavali M; Moradi A; Tahmaseb M; Mohammadian K; Ganji SM Circulating-Tumour DNA Methylation of HAND1 Gene: A Promising Biomarker in Early Detection of Colorectal Cancer. BMC Med. Genomics 2024, 17 (1), 117. 38689296 10.1186/s12920-024-01893-9 PMC11061902

[62] Xu T; Wang Q; Wang Q; Sun L Coupling High-Field Asymmetric Waveform Ion Mobility Spectrometry with Capillary Zone Electrophoresis-Tandem Mass Spectrometry for Top-Down Proteomics. Anal. Chem 2023, 95 (25), 9497–9504. 37254456 10.1021/acs.analchem.3c00551 PMC10540249

[63] Perez-Riverol Y; Csordas A; Bai J; Bernal-Llinares M; Hewapathirana S; Kundu DJ; Inuganti A; Griss J; Mayer G; Eisenacher M; Pérez E; Uszkoreit J; Pfeuffer J; Sachsenberg T; Yilmaz Ş; Tiwary S; Cox J; Audain E; Walzer M; Jarnuczak AF; Ternent T; Brazma A; Vizcaíno JA The PRIDE Database and Related Tools and Resources in 2019: Improving Support for Quantification Data. Nucleic Acids Res. 2019, 47 (D1), D442–D450. 30395289 10.1093/nar/gky1106 PMC6323896

