# Top-Down Proteomic Profiling of Protein Corona by High-Throughput Capillary Isoelectric Focusing-Mass Spectrometry

**DOI:** 10.1021/jasms.4c00463

**Published:** 2025-03-03

**Authors:** Reyhane Tabatabaeian Nimavard, Seyed Amirhossein Sadeghi, Morteza Mahmoudi, Guijie Zhu, Liangliang Sun

**Affiliations:** 1 Department of Chemistry, Michigan State University, 578 S Shaw Lane, East Lansing, Michigan 48824, United States; 2 Precision Health Program, Michigan State University, East Lansing, Michigan 48824, United States; 3 Department of Radiology, College of Human Medicine, Michigan State University, East Lansing, Michigan 48824, United States

**Keywords:** top-down proteomics, protein corona, cIEF-MS/MS, proteoform, nanomedicine

## Abstract

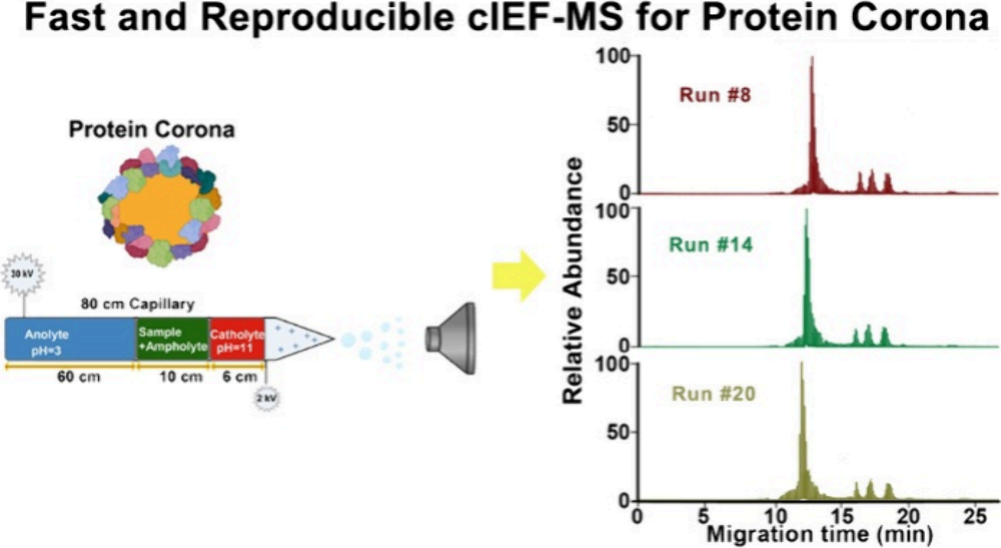

In the rapidly evolving field of nanomedicine, understanding the interactions between nanoparticles (NPs) and biological systems is crucial. A pivotal aspect of these interactions is the formation of a protein corona when NPs are exposed to biological fluids (e.g., human plasma), which significantly influences their behavior and functionality. This study introduces an advanced capillary isoelectric focusing tandem mass spectrometry (cIEF-MS/MS) platform designed to enable high-throughput and reproducible top-down proteomic analysis of protein corona. Our cIEF-MS/MS technique completed each analysis within 30 min. It produced reproducible proteoform measurements of protein corona for at least 50 runs regarding the proteoforms’ migration time [relative standard deviations (RSDs) <4%], the proteoforms’ intensity (Pearson’s correlation coefficients between any two runs >0.90), the number of proteoform identifications (71 ± 10), and the number of proteoform-spectrum matches (PrSMs) (196 ± 30). Of the 53 identified genes, 33 are potential biomarkers of various diseases (e.g., cancer, cardiovascular disease, and Alzheimer’s disease). We identified 1–102 proteoforms per potential protein biomarker, containing various sequence variations or post-translational modifications. Delineating proteoforms in protein corona by our cIEF-MS/MS in a reproducible and high-throughput fashion will benefit our understanding of nanobiointeractions and advance both diagnostic and therapeutic nanomedicine technologies.

## Introduction

In the past decade, extensive research has been conducted in the field of nanomedicine to achieve safer designs and more efficient therapeutic and diagnostic outcomes.^1,2^ To accomplish this, a deep understanding of the protein corona (a layer of biomolecules that adheres to the surfaces of nanoparticles (NPs) upon their interaction with biological fluids^3−7^) is essential. Comprehensive understanding of protein corona composition can substantially improve the capability of nanomedicine community to predict the way NPs interact with biosystems which in turn enables them to design safer and more efficient nanomedicines for both therapeutic and diagnostic (e.g., discovering novel protein biomarkers of diseases from human plasma) purposes.^8−11^

Mass spectrometry (MS)-based bottom-up proteomics (BUP) has been widely utilized to study the protein corona and generated rich information on the gene products and protein post-translational modifications (PTMs) in the protein corona.^12−14^ However, the BUP approach cannot provide information about the exact forms of protein molecules (“proteoforms”) in the protein corona due to the enzymatic digestion step. Proteoforms from the same gene can have divergent biological functions,^15,16^ and proteoforms in protein corona can significantly influence NP–cell interactions.^17,18^ Therefore, it is crucial to characterize the protein corona in a proteoform-specific manner.

MS-based top-down proteomics (TDP) is ideal for proteoform identification and quantification and has been widely used to study proteoform functions and discover proteoform biomarkers of diseases.^19−22^ We recently presented the first example of MS-based TDP for measuring the proteoforms in protein coronas by capillary zone electrophoresis (CZE)-MS.^23^ We also showed the advantages of capillary isoelectric focusing (cIEF)-MS for TDP of protein corona regarding the improved separation resolution and detection of large proteoforms compared to CZE-MS.^24^ Our previous CE-MS studies of protein corona required at least 1 h per run and did not provide long-term reproducibility investigations of CE-MS for TDP of protein coronas.

To establish a high-throughput, robust, and reproducible workflow for the broad application of MS-based top-down proteomics in protein corona analysis (aimed at advancing nanomedicine and discovering novel proteoform biomarkers of diseases), here we aim to develop an improved cIEF-MS/MS platform that enhances analysis throughput for top-down proteomic analysis of NP protein coronas with excellent reproducibility.

## Experimental Workflow

### Chemicals and Materials

The following materials were purchased from Sigma-Aldrich (St. Louis, MO): ammonium bicarbonate (ABC), 3-(trimethoxysilyl) propyl methacrylate (γ-MAPS), dithiothreitol (DTT), ammonia hydroxide (NH_3_H_2_O), ammonium acetate (NH_4_Ac), ammonium persulfate (APS), Pharmalytes with pI ranges of 3–10, 5–8 and 8–10.5 (GE Healthcare). HPLC-grade acetic acid (AA), MS-grade water, methanol (MeOH), formic acid (FA), Amicon Ultra (0.5 mL, 10 kDa cutoff size) centrifugal filter units, and fused silica capillaries (50 μm i.d./360 μm o.d., Polymicro Technologies) were purchased from Fisher Scientific (Pittsburgh, PA). Acrylamide was purchased from Acros Organics (Fair Lawn, NJ). A healthy human plasma sample was purchased from Innovative Research (www.innov-research.com) and diluted to 55% using phosphate buffer solution (PBS, 1X). Polystyrene NPs (PSNPs, ∼100 nm) were obtained from Polysciences (www.polysciences.com).

### Sample Preparation and Characterization

The sample preparation procedure for protein coronas is the same as for ref.^23,25^ Briefly, PSNPs were mixed with 55% human plasma. This mixture was stirred constantly for 1 h at a temperature of 37 °C to allow the formation of a protein corona. After an hour, the protein–NP complexes were separated by centrifugation at 14 000*g* for 20 min to remove unbound proteins. The resulting pellet was then washed twice with cold PBS.

Dynamic light scattering (DLS) analysis was performed to measure the size distribution of PSNPs before and after protein corona formation. The measurements were conducted at room temperature using a Zetasizer Nano Series DLS instrument (Malvern Instruments) equipped with a helium–neon laser at a wavelength of 632 nm.

For the collected protein corona coated PSNPS, the proteins were extracted from the NP surface by incubating the pellet in a 0.4% SDS solution with agitation for 1.5 h at 60 °C, and the extracted protein corona-containing supernatant was separated by centrifugation. An Amicon Ultra centrifugal filter with a 10 kDa molecular weight cutoff was used to exchange the buffer and remove the SDS. Finally, the protein corona sample in 100 mM ammonium bicarbonate (NH_4_HCO_3_) was measured using a BCA assay to determine the protein concentration, and it was adjusted to 1.5 mg/mL for MS analysis.

### cIEF-MS/MS Analysis

An automated cIEF-MS/MS system was built by combining a CESI 8000 Plus CE system (Beckman Coulter) with an Orbitrap Exploris 480 mass spectrometer (Thermo Fisher Scientific) using an in-house electrokinetically pumped sheath-flow CE-MS nanospray interface.^26^ The cIEF separation was carried out using an 80 cm long linear polyacrylamide (LPA)-coated capillary (50 μm i.d./360 μm o.d.).^27,28^ The LPA coating was made according to refs (27) and (28). One end of the separation capillary was etched using hydrofluoric acid to reduce its outer diameter to approximately 100 μm.^29^ The interface featured a glass spray emitter with an orifice size of 30–35 μm, filled with a sheath buffer composed of 0.2% (v/v) formic acid and 10% (v/v) methanol. The spray voltage was set to 2 kV, and the capillary outlet to emitter orifice distance was maintained at approximately 0.5 mm. The distance between the emitter orifice and the MS inlet was about 2 mm.

The automated cIEF-MS system was based on the “sandwich” injection approach.^30−32^ The injection sequence involved three steps: first, a 6 cm catholyte plug was injected at 10 psi for 8 s containing 0.3% NH_4_OH, followed by a 20 cm mixture of sample and ampholyte plug containing 0.6% ampholytes (3–10, 5–8, and 8–10.5, GE Healthcare), injected at 10 psi for 27 s. Approximately 600 ng of corona proteins (1.5 mg/mL, injection volume of 400 nL) were loaded into the capillary, and finally, a 50 cm anolyte plug was injected at 10 psi for 67 s containing 5% acetic acid. This combination provided efficient focusing and mobilization of the protein corona samples under a separation voltage of 30 kV.

The Orbitrap Exploris 480 mass spectrometer was used to analyze the proteoforms separated by cIEF in data-dependent acquisition (DDA) mode. Two approaches were used for data acquisition to detect both small and large (>30 kDa) proteoforms. For small proteoforms (<30 kDa), we employed a “high-resolution MS1 and high-resolution MS/MS” mode, i.e., “High–High” mode. The detailed parameters for the “High–High” mode include MS1 resolution 480,000 at *m*/*z* 200 with a single microscan across a *m*/*z* range of 700–3000. Maximum ion injection time was set to 50 ms for MS and 100 ms for MS/MS. Normalized AGC target 300%, Ions with an intensity of over 1E4 and charge states varying from 5 to 60 were isolated with a 2 *m*/*z* window, followed by fragmentation through higher-energy collision dissociation (HCD) at 25% normalized collision energy (NCE). Dynamic exclusion was enabled with a duration of 30 s and a mass tolerance of 10 ppm, and isotope exclusion was activated. The fragment ions were detected with a resolution of 120 000 at *m*/*z* 200 and normalized AGC 100%. For large proteoforms (>30 kDa), a “low-resolution MS1 and high-resolution MS/MS” mode, i.e., “Low–High” mode, was employed. MS1 resolution of 7,500 at *m*/*z* 200 was used. The microscan setting is 3. The other parameters are the same as the “High–High” mode.

### Data Analysis

Data processing was conducted using the TopPIC software developed by Liu’s group to identify and quantify proteoforms in the “High–High” mode.^33^ For the “Low–High” mode, the UniDec software facilitated mass deconvolution,^34^ determining the average masses of larger proteoforms. The cIEF-MS/MS data analysis began with converting RAW files to the mzML format using MSconvert. The converted data was then processed using TopFD (version 1.7.0) software^35^ to convert isotope clusters into monoisotopic masses and identifiable proteoform features, with the results stored in msalign and text files. The deconvoluted mass spectra and proteoform features were then searched against a home-built protein database of approximately 1,000 sequences using TopPIC software (version 1.7.0), which included proteins previously identified in bottom-up proteomics (BUP) data. TopPIC was configured to accommodate a single unexpected mass shift per proteoform with a maximum shift of 500 Da and maintained a mass error tolerance of 50 ppm for both precursor and fragment ions. A target-decoy approach was used to estimate and control the false discovery rate (FDR), setting it at 1% at the proteoform-spectrum match (PrSM) level and 5% at the proteoform level. Finally, the identified proteoforms were quantified using TopDiff software to enable label-free quantification across technical replicates. The quantification aggregated the intensities of each proteoform’s peaks across all scans and charge states.^36^ The raw mass spectrometry data files were processed using an Xcalibur Qual Browser (Thermo Fisher Scientific) to extract proteoform intensity values and migration time information. Base peak chromatograms and extracted ion chromatograms were generated to visualize the separation profiles. The electropherograms are graphically refined using Adobe Illustrator for figure preparation.

## Results and Discussion

**Figure 1:**
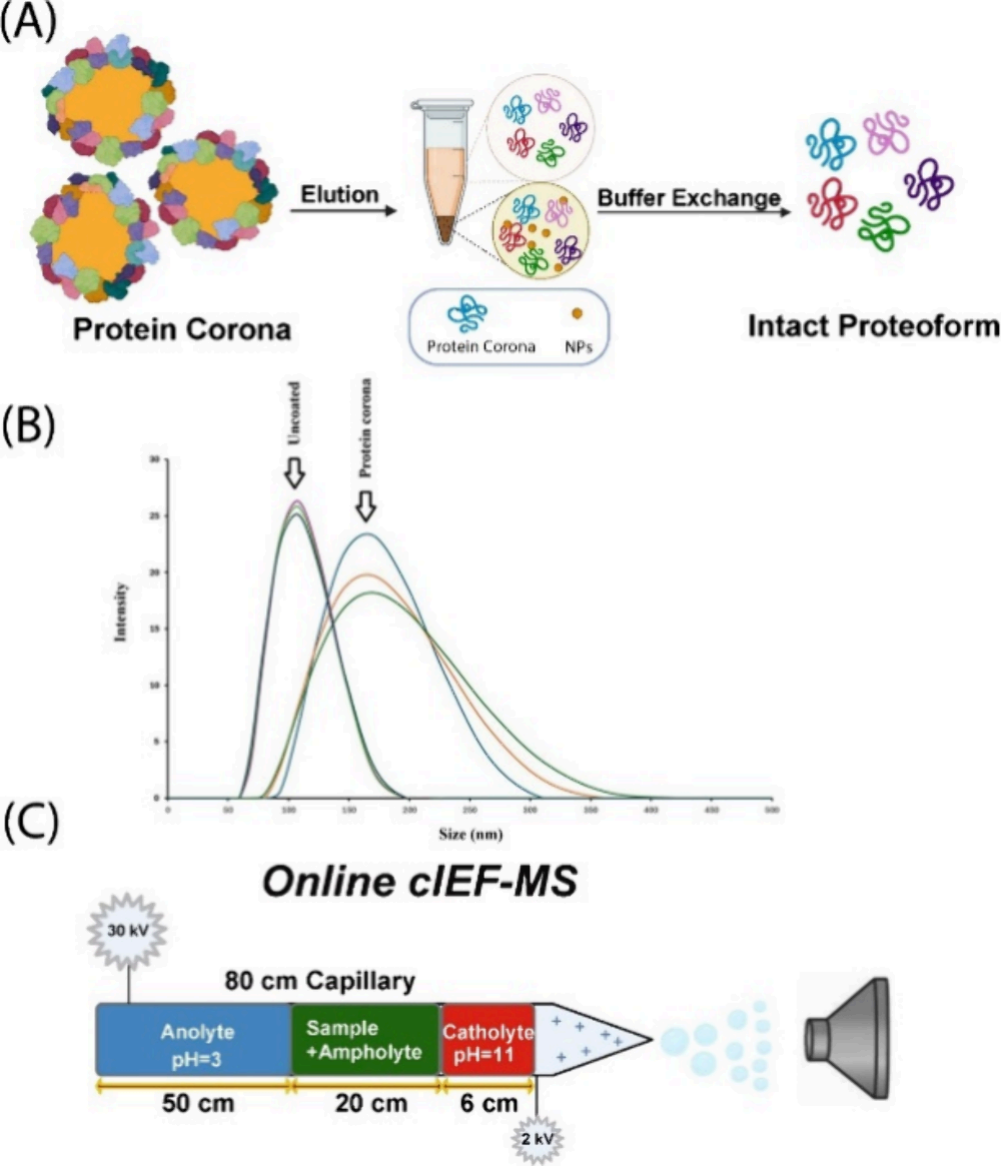
Schematic workflow of TDP of protein coronas using the advanced cIEF-MS/MS technique. (A) Brief workflow of preparing the protein corona sample for TDP after incubating the PSNPs with a human plasma sample to form the protein corona on the surface of PSNPs. (B) DLS analysis of bare PSNPs (uncoated) and protein corona coated NPs (protein corona). (C) Schematic design of the high-throughput cIEF-MS/MS for protein corona analysis.

We developed a high-throughput automated cIEF-MS/MS technique that took 30 min or less per run and applied it to the TDP of NP protein coronas (Figure 1). The protein corona sample was prepared according to our previous procedure.^29^ Briefly, proteoforms in the protein corona of PSNPs were eluted using a 0.4% SDS buffer and cleaned up by buffer exchange, followed by cIEF-MS/MS, Figure 1A. DLS analysis revealed protein corona formation at the surface of PSNPs, evidenced by increasing the size of corona-coated NPs, Figure 1B. The advanced cIEF-MS/MS technique for high-throughput TDP analysis of protein coronas was carried out by employing a short separation capillary for cIEF-MS with a commercial CE system, Figure 1C. An 80 cm long LPA-coated capillary was used, and the effective capillary length for cIEF separation was shorter than 30 cm because we used a “sandwich” injection approach, injecting a plug of catholyte (0.3% NH_4_OH, pH ∼ 11), a plug of the sample with ampholyte in 100 mM NH_4_HCO_3_, and a long plug of anolyte (5% acetic acid, pH 2.4). We determined the optimal conditions as a 6 cm plug of catholyte, a 20 cm sample plug containing 0.6% ampholytes (pI 3–10, 5–8, and 8–10.5 with ratios 1:1:1), and a 50 cm plug of anolyte using a standard protein mixture (data not shown). Because of the short effective capillary length for cIEF (≤30 cm), the analysis could be carried out in a high-throughput fashion. Also, because the total capillary length was 80 cm, a regular commercial CE system could be used, allowing the technique to be easily adopted by other researchers.

### Reproducibility of High-Throughput cIEF-MS/MS-Based TDP for Protein Corona

**Figure 2:**
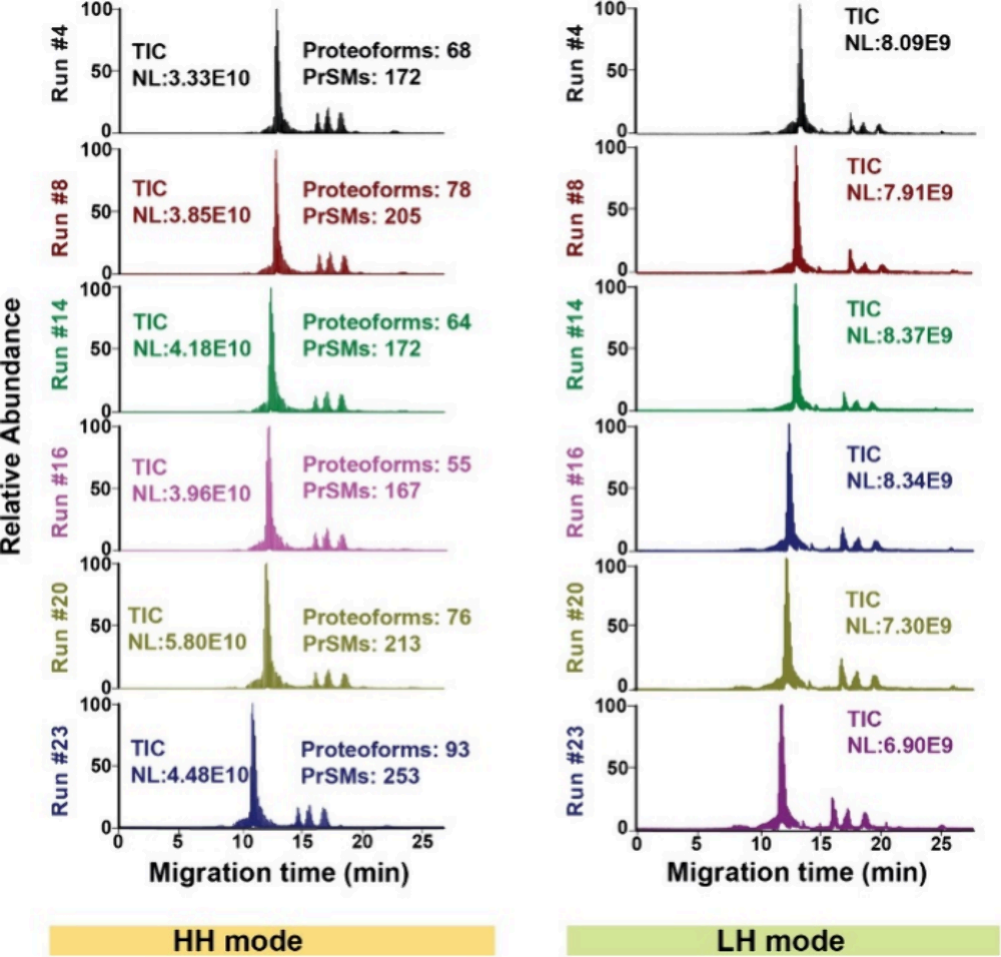
Total ion current (TIC) electropherograms of protein corona proteoforms as determined by CIEF-MS/MS in “High–High (HH)” and “Low–High (LH)” modes. Six selected electropherograms from runs #4, #8, #14, #16, #20, and #23 in HH and LH modes are shown.

We analyzed the protein corona sample of PSNPs using the optimal high-throughput cIEF-MS/MS technique for 50 runs, Figure 2. The 50 runs were performed on the same corona sample to evaluate the technical reproducibility of our cIEF-MS/MS method. Each cIEF-MS run took less than 30 min, producing a 2- to 6-fold improvement in analysis throughput compared to the previous cIEF-MS/MS-based TDP studies of complex samples.^24,26,30,37,38^ We performed 25 runs in the “High–High” mode and 25 runs in the “Low–High” mode to evaluate the technique for both small and large proteoform measurements.

**Figure 3:**
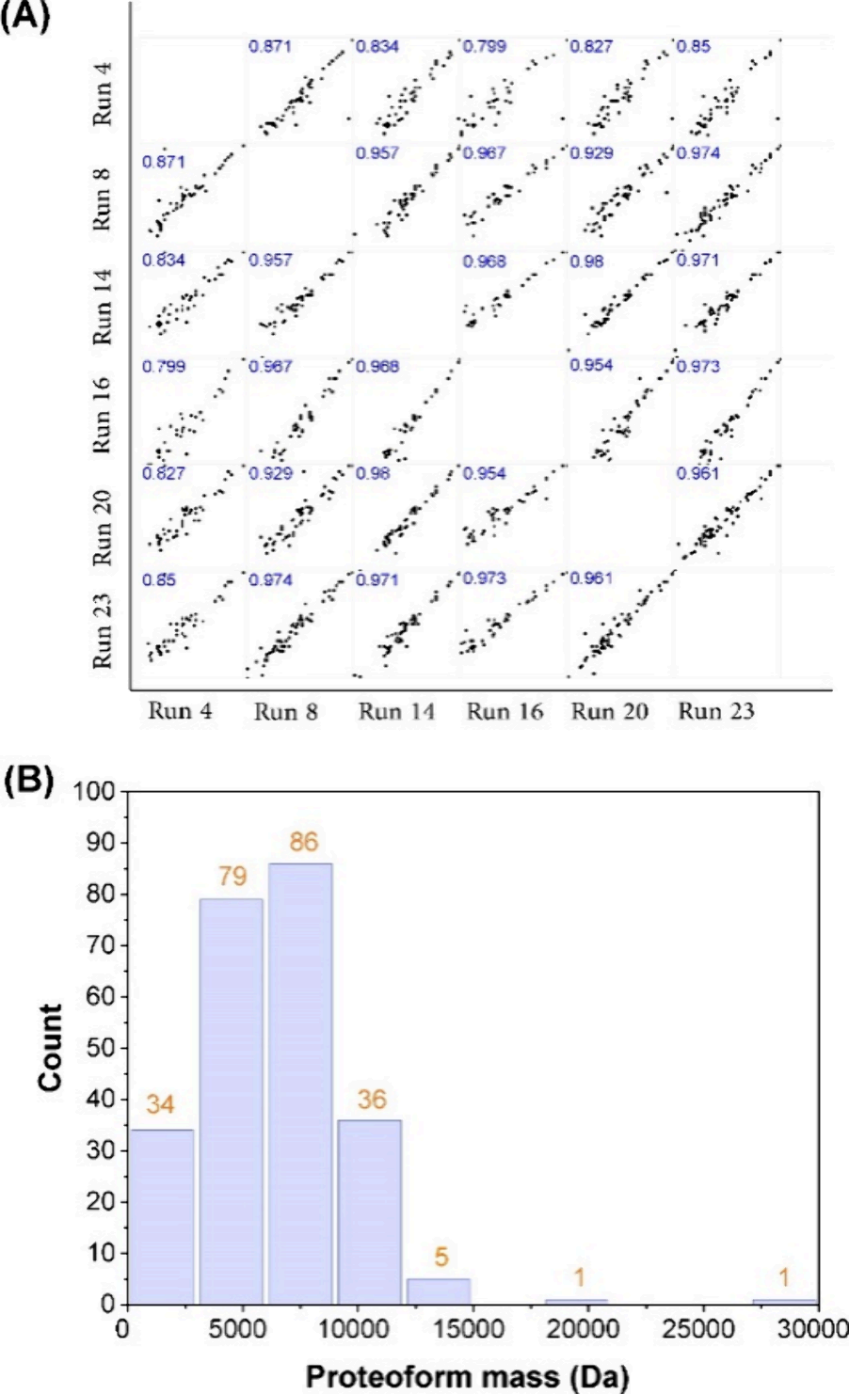
(A) Intensity correlations of overlapped proteoforms between any two cIEF-MS/MS runs. Six runs were randomly selected for this analysis. Proteoform intensities were log 2 transformed for the plot, and Pearson’s correlation coefficient (*r*) values were labeled. (B) Mass distribution of the identified proteoforms from 25 cIEF-MS/MS runs (High–High).

The cIEF-MS/MS technique produced reproducible separation, detection, and identification of proteoforms. The electropherograms in Figure 2A,B show consistent separation profiles of proteoforms in both “High–High” and “Low–High” modes. In the “High–High” mode, a normalized level (NL) of 4.0 ± 0.8 E10 (*n* = 25) was obtained for the total ion current (TIC) electropherograms, corresponding to a relative standard deviation (RSD) of 20%. In the “Low–High” mode, the NL was 8.2 ± 0.8E09, corresponding to an RSD of about 10%. The numbers of proteoform and proteoform-spectrum match (PrSM) identifications are also consistent across the “High–High” runs, with 71 ± 10 (*n* = 25) proteoforms and 196 ± 30 (*n* = 25) PrSMs. Importantly, the new cIEF-MS/MS technique produced a similar number of proteoform identifications to our previous cIEF-MS/MS technique for the same protein corona sample^24^ (∼70 vs ∼60) but with a 2-fold improvement in analysis throughput (30 vs 60 min). In the “Low–High” runs, three large proteins (28, 51, and 66 kDa) with multiple proteoforms per protein were consistently detected, Figure S1 in Supporting Information. Those three proteins correspond to human serum albumin (HSA, 66 kDa), apolipoprotein A-I (APOA1, 28 kDa), and an unknown protein (51 kDa). The data agreed reasonably with our previous results.^24,25^ We randomly selected seven proteoforms from seven genes and determined their migration times across the 25 “High–High” runs from the database search results to further evaluate the separation reproducibility, Table 1. The RSDs of the migration time of those proteoforms are less than 4% across 25 cIEF-MS/MS runs, indicating excellent reproducibility of the technique for proteoform separation. To validate the consistency of the high-throughput cIEF-MS/MS methodology for protein corona analysis regarding proteoform intensity, we randomly chose six cIEF-MS/MS runs (“High–High”) and plotted the intensity of overlapped proteoforms between any two runs, Figure 3A. The proteoform intensity showed strong linear correlations between runs, evidenced by the high Pearson’s correlation coefficient (*r*) of 0.92 ± 0.06, underscoring the quantitative reproducibility of the TDP technique for protein corona analysis. Figure 3B shows the mass distribution of identified proteoforms from all of the “High–High” runs. The mass of identified proteoforms ranged from ∼2 kDa to ∼30 kDa, and the majority of them were ∼10 kDa or smaller. The identified proteoforms from each “High–High” run are listed in the Supporting Information xlsx file. We must point out that if we include the large proteoforms detected in “Low–High” mode, the mass range of identified proteoforms will be extended to 2–66 kDa.

**Table 1:** Summary of Migration Times of Seven Selected Proteoforms from Seven Proteins across 25 “High–High” Runs

protein	migration time (mean, s)	standard deviation (SD, s)	relative standard deviation (RSD, %)
Apolipoprotein E	899.81	19.8	2.2
Apolipoprotein A-II	881.8	22.0	2.5
Apolipoprotein C-II	1008.6	34.4	3.4
Apolipoprotein A-I	949.8	22.2	2.3
Fibrinogen α chain	806.0	19.3	2.4
Apolipoprotein C-III	1589.3	62.4	3.9
Clusterin	824.1	24.4	3.0

### Protein Biomarkers Identified by cIEF-MS/MS Analysis of Protein Corona

**Figure 4:**
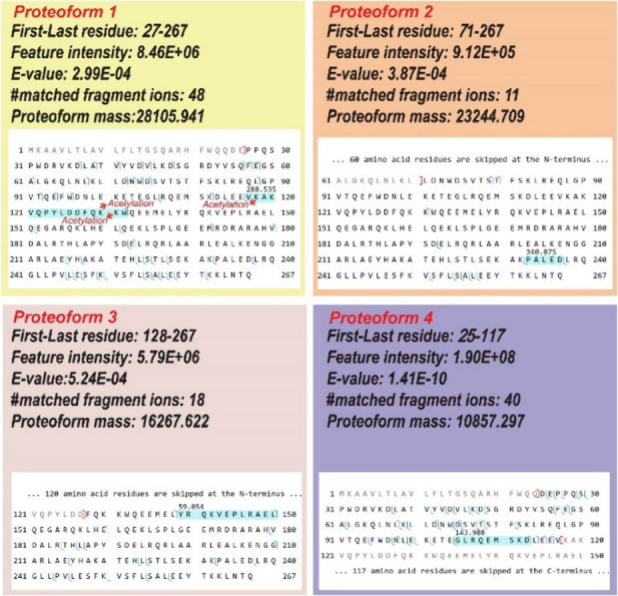
Sequences and fragmentation patterns of four distinct proteoforms of apolipoprotein A-I (APOA1) identified using cIEF-MS/MS-based TDP in “High–High” mode.

Our TDP analysis of the protein corona identified 53 genes, and the number of detected proteoforms per gene ranged from 1 to 102, Table 2. 33 out of the 53 genes are biomarkers, and they span various protein families and functional classes, including but not limited to apolipoproteins, complement proteins, immunoglobulins, and cytoskeletal proteins. Many of these proteins are associated with diverse diseases and pathological states, underscoring their potential utility as diagnostic or prognostic biomarkers.^39−43^ Particularly noteworthy is the prominence of the apolipoprotein family within the data set, which includes APOA1, APOA2, APOA4, APOB, APOC2, APOC3, APOE, and APOF. These proteins play critical roles in lipid metabolism and are strongly linked to cardiometabolic disorders such as dyslipidemia, metabolic syndrome, atherosclerosis, and cardiovascular diseases.^44−46^ This connection provides a significant opportunity for further research into their pathobiological mechanisms and applications in clinical diagnostics.^47−49^ Multiple proteoforms were identified for most of the apolipoprotein family members. For example, we identified 102 proteoforms of the *APOA1* gene. Nearly 90% are N-terminally truncated, and only several proteoforms have C-terminal truncations. About 85% of proteoforms carry mass shifts due to sequence variations or modifications. Four *APOA1* proteoforms are shown in Figure 4. Those proteoforms carry variations due to signal peptide cleavages, truncations, and PTMs. Proteoform 1 has an N-terminal cleavage of the first 26 amino acid residues, most likely corresponding to signal peptide cleavage. Proteoform 1 also contains a mass shift of +288.535 Da in the highlighted region. Based on the PTM information in the dbPTM database (https://awi.cuhk.edu.cn/dbPTM/),^50^ three lysine residues in the highlighted region can be acetylated, corresponding to a +126 Da mass shift. The +288.535 Da value may correspond to the combination of acetylation and other PTMs. For proteoform 2, the first 70 amino acid residues were truncated, and it carries a mass shift of +340.875 Da. Proteoform 3 shows a truncation of the first 127 amino acid residues at the N-terminus. This proteoform also contains an unknown mass shift of +59.054 Da in the highlighted region. The exact nature of this modification is required for further investigation. Proteoform 4 exhibits an N-terminal removal of the first 24 amino acid residues due to signal peptide cleavage and a C-terminal truncation. This proteoform also contains an unknown mass shift of +143.988 Da in the highlighted region. Additionally, the data set identifies biomarkers pertinent to inflammatory and autoimmune diseases, including complement proteins (C3, C9),^51−53^ serum amyloid A proteins (SAA1), and serpins (SERPINA1, SERPINC1).^51,54^ The catalog also highlights biomarkers associated with neurodegenerative conditions such as Alzheimer’s disease (APOE, CLU)^55,56^ and Parkinson’s disease (ABCB9),^57^ as well as proteins involved in cancer progression and metastasis, such as ACTB, KRT1, and RAB15.^58−61^ These findings demonstrate that TDP studies of protein coronas from large cohorts of plasma samples with various diseases using our high-throughput cIEF-MS/MS technique could discover novel proteoform biomarkers of diseases, facilitating disease early diagnosis and drug development.

**Table 2:** Summary of the Identified Genes and Corresponding Number of Proteoforms from the cIEF-MS/MS-Based TDP Analysis of Nanoparticle Protein Coronas

gene name	no. of proteoforms	protein description	biomarker
ACTB	**1**	Actin beta	cancer, neurological disorders, and cardiovascular diseases.
ACTG2 (F8WCH0)	1	Actin gamma 2, smooth muscle	
ALB	1	Albumin	cardiovascular diseases, liver function, inflammation, and malnutrition.
SERPINA1 (A1AT)	9	Alpha-1-antitrypsin	lung diseases, such as chronic obstructive pulmonary disease (COPD) and emphysema.
SERPINA1 (G3V387)	1	Alpha-1-antitrypsin (fragment)	
SERPINA2 (A1ATR)	1	Alpha-1-antitrypsin-related protein	
APOA1	102	Apolipoprotein A-I	cardiovascular diseases, diabetes, and certain types of cancer
APOA2	24	Apolipoprotein A-II	cardiometabolic conditions such as dyslipidemia, metabolic syndrome, and atherosclerosis.
APOA2 (V9GYM3)	7	Apolipoprotein A-II	
APOA2 (V9GYS1)	20	Apolipoprotein A-II	
APOA2 (V9GYC1)	1	Apolipoprotein A-II (fragment)	
APOA4	8	Apolipoprotein A-IV	cardiometabolic conditions such as dyslipidemia, metabolic syndrome, and atherosclerosis.
APOB	14	Apolipoprotein B-100	cardiovascular diseases, such as atherosclerosis and heart disease.
APOC2	30	Apolipoprotein C-II	cardiometabolic conditions such as dyslipidemia, metabolic syndrome, and atherosclerosis.
APOC2 (V9GYJ8)	14	Apolipoprotein C-II	
APOC2 (Q6P163)	2	Apolipoprotein C-II	
APOC3	43	Apolipoprotein C-III	cardiovascular diseases and metabolic disorders, such as hypertriglyceridemia
APOC3 (BOYIW2)	4	Apolipoprotein C-III	
APOC3 (C9J2Q0)	3	Apolipoprotein C-III (fragment)	
APOE	14	Apolipoprotein E	Alzheimer’s disease and other neurodegenerative disorders.
APOF	1	Apolipoprotein F	cardiometabolic conditions such as dyslipidemia, metabolic syndrome, and atherosclerosis.
APOL1	1	Apolipoprotein L1	kidney disease, especially in high-risk populations.
SERPINC1 (ANT3)	1	Apolipoprotein L1	
ABCB9	1	ATP binding cassette subfamily B member 9 (fragment)	Parkinson’s disease and Alzheimer’s disease.
CPN2	1	Carboxypeptidase N subunit 2	sepsis and acute pancreatitis.
CLU	3	Clusterin	neurodegenerative disorders, cancer, and kidney disease.
C1R	1	Complement C1r (fragment)	may have potential for the systemic lupus erythematosus and rheumatoid arthritis
C1RL	1	Complement C1r subcomponent like (fragment)	may have potential for inflammatory and autoimmune disorders, such as systemic lupus erythematosus and rheumatoid arthritis
C3	15	Complement C3	inflammatory and autoimmune diseases, such as rheumatoid arthritis and systemic lupus erythematosus.
C9	1	Complement C9	inflammatory and autoimmune diseases, such as systemic lupus erythematosus and rheumatoid arthritis.
FETUB	1	Fetuin B (Fragment)	may have potential for metabolic disorders, such as chronic kidney disease and nonalcoholic fatty liver disease (NAFLD)
FGA	7	Fibrinogen alpha chain	cardiovascular diseases, thrombosis, and inflammation.
FGB	7	Fibrinogen beta chain	
FGG	5	Fibrinogen gamma chain	
GSN	1	Gelsolin	Alzheimer’s disease, amyloidosis, and critical illness
PLG	1	HCG2029799, isoform CRA_d	
IGLV2-8	1	Immunoglobulin lambda variable 2–8	
IGLL5	1	Immunoglobulin lambda-like polypeptide 5	hematological cancers.
ITIH1	4	Interalpha-trypsin inhibitor heavy chain	cancer, liver disease, and inflammatory conditions
ITIH4	3	Interalpha-trypsin inhibitor heavy chain H4	
KRT1	1	Keratin, type II cytoskeletal 1	epithelial cancers, such as lung, breast, and prostate cancer.
KRT71	1	Keratin, type II cytoskeletal 71	
KRT74	1	Keratin, type II cytoskeletal 74	
DEFA1	1	Neutrophil defensin 1	infection severity such as acute respiratory distress syndrome (ARDS) and sepsis.
RAB15	1	RAB15, member RAS oncogene family	certain cancers, such as lung, pancreatic, and colorectal cancer.
RAP1B	1	RAP1B, member of RAS oncogene family (fragment)	may have potential for certain cancers, such as lung, pancreatic, and colorectal cancer.
SELENOP	1	Selenoprotein P (fragment)	may have potential for various conditions, including oxidative stress, inflammation, and metabolic disorders.
SAA1 (E9PQD6)	6	Serum amyloid A protein	May have potential for various inflammatory conditions and neoplastic disorders, such as rheumatoid arthritis, Crohn’s disease, and certain types of cancer.
SAA1	14	Serum amyloid A-1 protein	various inflammatory conditions, such as rheumatoid arthritis, Crohn’s disease, and certain types of cancer.
SHBG	1	Sex hormone binding globulin	
TTR	7	Transthyretin	amyloidosis, a group of diseases characterized by the accumulation of abnormal proteins in tissues.
TTR (A0A087WT59)	2	Transthyretin	
TUBA1C	1	Tubulin alpha chain	cancer, neurodegenerative disorders, and certain types of ciliopathies.

## Conclusions

A high-throughput cIEF-MS/MS technique was developed to profile the proteoform composition of protein coronas with excellent qualitative and quantitative reproducibility across 50 runs. The number of proteoform identifications per cIEF-MS/MS run for protein coronas is comparable with our previous cIEF-MS/MS data^24,25^ but with a 2-fold improvement in analysis throughput (30 min per run vs 60 min per run). Our advanced cIEF-MS/MS technique is ready for quantitative TDP analysis of large cohorts of human plasma samples using the protein corona approach to discover novel proteoform biomarkers of various diseases.

We also must note that the number of proteoform identifications from our high-throughput cIEF-MS/MS is lower than that produced by CZE-MS/MS^23^ for the same protein corona sample (∼70 vs ∼100), although cIEF-MS/MS has a 2-fold higher analysis throughput than CZE-MS/MS (30 vs 60 min per run). The reason is mainly due to the significant ionization suppression of proteoforms from high-concentration ampholytes in cIEF-MS/MS. We expect that integrating FAIMS (high field asymmetric waveform ion mobility spectrometry)^62^ with our fast cIEF-MS/MS may be useful to advance the technique further toward better proteoform coverage of the protein corona samples because FAIMS can potentially separate ampholytes from proteoforms in the gas phase due to their substantial difference in size.

## Data Availability

The MS RAW files about TDP measurement were deposited to the ProteomeXchange Consortium via PRIDE, with the data set identifier PXD052764.^63^

## Supporting Information Available

Mass spectra and deconvoluted mass spectra of three large proteins detected in the “low–high” runs (PDF)
Lists of proteoforms identified from each cIEF-MS/MS run in “High–High” mode (XLSX)

## References

[ref1] Pelaz B.; Alexiou C.; Alvarez-Puebla R. A.; Alves F.; Andrews A. M.; Ashraf S.; Balogh L. P.; Ballerini L.; Bestetti A.; Brendel C.; Bosi S.; Carril M.; Chan W. C. W.; Chen C.; Chen X.; Chen X.; Cheng Z.; Cui D.; Du J.; Dullin C.; Escudero A.; Feliu N.; Gao M.; George M.; Gogotsi Y.; Grünweller A.; Gu Z.; Halas N. J.; Hampp N.; Hartmann R. K.; Hersam M. C.; Hunziker P.; Jian J.; Jiang X.; Jungebluth P.; Kadhiresan P.; Kataoka K.; Khademhosseini A.; Kopeček J.; Kotov N. A.; Krug H. F.; Lee D. S.; Lehr C. M.; Leong K. W.; Liang X. J.; Lim M. L.; Liz-Marzán L. M.; Ma X.; Macchiarini P.; Meng H.; Möhwald H.; Mulvaney P.; Nel A. E.; Nie S.; Nordlander P.; Okano T.; Oliveira J.; Park T. H.; Penner R. M.; Prato M.; Puntes V.; Rotello V. M.; Samarakoon A.; Schaak R. E.; Shen Y.; Sjöqvist S.; Skirtach A. G.; Soliman M. G.; Stevens M. M.; Sung H. W.; Tang B. Z.; Tietze R.; Udugama B. N.; VanEpps J. S.; Weil T.; Weiss P. S.; Willner I.; Wu Y.; Yang L.; Yue Z.; Zhang Q.; Zhang Q.; Zhang X. E.; Zhao Y.; Zhou X.; Parak W. J. Diverse Applications of Nanomedicine. ACS Nano 2017, 11 (3), 2313–2381. 10.1021/acsnano.6b06040. 28290206 PMC5371978

[ref2] Faria M.; Björnmalm M.; Thurecht K. J.; Kent S. J.; Parton R. G.; Kavallaris M.; Johnston A. P. R.; Gooding J. J.; Corrie S. R.; Boyd B. J.; Thordarson P.; Whittaker A. K.; Stevens M. M.; Prestidge C. A.; Porter C. J. H.; Parak W. J.; Davis T. P.; Crampin E. J.; Caruso F. Minimum Information Reporting in Bio-Nano Experimental Literature. Nat. Nanotechnol 2018, 13 (9), 777–785. 10.1038/s41565-018-0246-4. 30190620 PMC6150419

[ref3] Mitchell M. J.; Billingsley M. M.; Haley R. M.; Wechsler M. E.; Peppas N. A.; Langer R. Engineering Precision Nanoparticles for Drug Delivery. Nat. Rev. Drug Discovery 2021, 20, 101–124. 10.1038/s41573-020-0090-8. 33277608 PMC7717100

[ref4] Sadeghi A.; PourEskandar S.; Askari E.; Akbari M. Polymeric Nanoparticles and Nanogels: How Do They Interact with Proteins?. Gels 2023, 9, 632 10.3390/gels9080632. 37623087 PMC10453451

[ref5] Hajipour M. J.; Laurent S.; Aghaie A.; Rezaee F.; Mahmoudi M. Personalized Protein Coronas: A “Key” Factor at the Nanobiointerface. Biomater Sci. 2014, 2 (9), 1210–1221. 10.1039/C4BM00131A. 32481892

[ref6] Patra J. K.; Das G.; Fraceto L. F.; Campos E. V. R.; Rodriguez-Torres M. D. P.; Acosta-Torres L. S.; Diaz-Torres L. A.; Grillo R.; Swamy M. K.; Sharma S.; Habtemariam S.; Shin H. S. Nano Based Drug Delivery Systems: Recent Developments and Future Prospects. J. Nanobiotechnol. 2018, 16, 71 10.1186/s12951-018-0392-8. PMC6145203 30231877

[ref7] Bertrand N.; Grenier P.; Mahmoudi M.; Lima E. M.; Appel E. A.; Dormont F.; Lim J. M.; Karnik R.; Langer R.; Farokhzad O. C. Mechanistic Understanding of in Vivo Protein Corona Formation on Polymeric Nanoparticles and Impact on Pharmacokinetics. Nat. Commun. 2017, 8 (1), 777 10.1038/s41467-017-00600-w. 28974673 PMC5626760

[ref8] Walczyk D.; Bombelli F. B.; Monopoli M. P.; Lynch I.; Dawson K. A. What the Cell “Sees” in Bionanoscience. J. Am. Chem. Soc. 2010, 132 (16), 5761–5768. 10.1021/ja910675v. 20356039

[ref9] Corbo C.; Li A. A.; Poustchi H.; Lee G. Y.; Stacks S.; Molinaro R.; Ma P.; Platt T.; Behzadi S.; Langer R.; Farias V.; Farokhzad O. C. Analysis of the Human Plasma Proteome Using Multi-Nanoparticle Protein Corona for Detection of Alzheimer’s Disease. Adv. Healthcare Mater. 2021, 10 (2), 2000948 10.1002/adhm.202000948. 33169521

[ref10] Mahmoudi M.; Landry M. P.; Moore A.; Coreas R. The Protein Corona from Nanomedicine to Environmental Science. Nat. Rev. Mater. 2023, 8, 422–438. 10.1038/s41578-023-00552-2. PMC10037407 37361608

[ref11] Ren J.; Cai R.; Wang J.; Daniyal M.; Baimanov D.; Liu Y.; Yin D.; Liu Y.; Miao Q.; Zhao Y.; Chen C. Precision Nanomedicine Development Based on Specific Opsonization of Human Cancer Patient-Personalized Protein Coronas. Nano Lett. 2019, 19 (7), 4692–4701. 10.1021/acs.nanolett.9b01774. 31244235

[ref12] Ashkarran A. A.; Gharibi H.; Voke E.; Landry M. P.; Saei A. A.; Mahmoudi M. Measurements of Heterogeneity in Proteomics Analysis of the Nanoparticle Protein Corona across Core Facilities. Nat. Commun. 2022, 13 (1), 6610 10.1038/s41467-022-34438-8. 36329043 PMC9633814

[ref13] Jiang Y.; Rex D. A. B.; Schuster D.; Neely B. A.; Rosano G. L.; Volkmar N.; Momenzadeh A.; Peters-Clarke T. M.; Egbert S. B.; Kreimer S.; Doud E. H.; Crook O. M.; Yadav A. K.; Vanuopadath M.; Hegeman A. D.; Mayta M. L.; Duboff A. G.; Riley N. M.; Moritz R. L.; Meyer J. G. Comprehensive Overview of Bottom-Up Proteomics Using Mass Spectrometry. ACS Measur. Sci. Au 2024, 4, 338–417. 10.1021/acsmeasuresciau.3c00068. PMC11348894 39193565

[ref14] Miller R. M.; Smith L. M. Overview and Considerations in Bottom-up Proteomics. Analyst 2023, 148, 475–486. 10.1039/D2AN01246D. 36383138 PMC9898146

[ref15] Smith L. M.; Agar J. N.; Chamot-Rooke J.; Danis P. O.; Ge Y.; Loo J. A.; Paša-Tolić L.; Tsybin Y. O.; Kelleher N. L.; Consortium for Top-Down Proteomics. Human Proteoform Project: Defining the human proteome. Sci. Adv. **2021**, 7, eabk0734. 34767442 10.1126/sciadv.abk0734 PMC8589312

[ref16] Smith L. M.; Kelleher N. L. Proteoforms as the next Proteomics Currency. Science 2018, 359 (6380), 1106–1107. 10.1126/science.aat1884. 29590032 PMC5944612

[ref17] Saei A. A.; Sun L.; Mahmoudi M. The Role of Protein Corona in Advancing Plasma Proteomics. Proteomics 2025, 25, e2400028 10.1002/pmic.202400028. 39221533 PMC11735278

[ref18] Blume J. E.; Manning W. C.; Troiano G.; Hornburg D.; Figa M.; Hesterberg L.; Platt T. L.; Zhao X.; Cuaresma R. A.; Everley P. A.; Ko M.; Liou H.; Mahoney M.; Ferdosi S.; Elgierari E. M.; Stolarczyk C.; Tangeysh B.; Xia H.; Benz R.; Siddiqui A.; Carr S. A.; Ma P.; Langer R.; Farias V.; Farokhzad O. C. Rapid, Deep and Precise Profiling of the Plasma Proteome with Multi-Nanoparticle Protein Corona. Nat. Commun. 2020, 11 (1), 3662 10.1038/s41467-020-17033-7. 32699280 PMC7376165

[ref19] Chen B.; Brown K. A.; Lin Z.; Ge Y. Top-Down Proteomics: Ready for Prime Time?. Anal. Chem. 2018, 90, 110–127. 10.1021/acs.analchem.7b04747. 29161012 PMC6138622

[ref20] Tran J. C.; Zamdborg L.; Ahlf D. R.; Lee J. E.; Catherman A. D.; Durbin K. R.; Tipton J. D.; Vellaichamy A.; Kellie J. F.; Li M.; Wu C.; Sweet S. M. M.; Early B. P.; Siuti N.; Leduc R. D.; Compton P. D.; Thomas P. M.; Kelleher N. L. Mapping Intact Protein Isoforms in Discovery Mode Using Top-down Proteomics. Nature 2011, 480 (7376), 254–258. 10.1038/nature10575. 22037311 PMC3237778

[ref21] Donnelly D. P.; Rawlins C. M.; DeHart C. J.; Fornelli L.; Schachner L. F.; Lin Z.; Lippens J. L.; Aluri K. C.; Sarin R.; Chen B.; Lantz C.; Jung W.; Johnson K. R.; Koller A.; Wolff J. J.; Campuzano I. D. G.; Auclair J. R.; Ivanov A. R.; Whitelegge J. P.; Paša-Tolić L.; Chamot-Rooke J.; Danis P. O.; Smith L. M.; Tsybin Y. O.; Loo J. A.; Ge Y.; Kelleher N. L.; Agar J. N. Best Practices and Benchmarks for Intact Protein Analysis for Top-down Mass Spectrometry. Nat. Methods 2019, 16 (7), 587–594. 10.1038/s41592-019-0457-0. 31249407 PMC6719561

[ref22] Catherman A. D.; Durbin K. R.; Ahlf D. R.; Early B. P.; Fellers R. T.; Tran J. C.; Thomas P. M.; Kelleher N. L. Large-Scale Top-down Proteomics of the Human Proteome: Membrane Proteins, Mitochondria, and Senescence. Mol. Cell. Proteomics 2013, 12 (12), 3465–3473. 10.1074/mcp.M113.030114. 24023390 PMC3861700

[ref23] Sadeghi S. A.; Ashkarran A. A.; Wang Q.; Zhu G.; Mahmoudi M.; Sun L. Mass Spectrometry-Based Top-Down Proteomics in Nanomedicine: Proteoform-Specific Measurement of Protein Corona. ACS Nano 2024, 18 (38), 26024–26036. 10.1021/acsnano.4c04675. 39276099 PMC11440641

[ref24] Zhu G.; Sadeghi S. A.; Mahmoudi M.; Sun L. Deciphering Nanoparticle Protein Coronas by Capillary Isoelectric Focusing-Mass Spectrometry-Based Top-down Proteomics. Chem. Commun. 2024, 60 (81), 11528–11531. 10.1039/D4CC02666G. PMC11418007 39310940

[ref25] Sadeghi S. A.; Ashkarran A. A.; Mahmoudi M.; Sun L. Mass spectrometry-based top-down proteomics in nanomedicine: proteoform-specific measurement of protein corona. bioRxiv 2024, 10.1101/2024.03.22.586273. PMC11440641 39276099

[ref26] Xu T.; Shen X.; Yang Z.; Chen D.; Lubeckyj R. A.; McCool E. N.; Sun L. Automated Capillary Isoelectric Focusing-Tandem Mass Spectrometry for Qualitative and Quantitative Top-Down Proteomics. Anal. Chem. 2020, 92 (24), 15890–15898. 10.1021/acs.analchem.0c03266. 33263984 PMC8564864

[ref27] Chen D.; Shen X.; Sun L. Capillary Zone Electrophoresis-Mass Spectrometry with Microliter-Scale Loading Capacity, 140 min Separation Window and High Peak Capacity for Bottom-up Proteomics. Analyst 2017, 142 (12), 2118–2127. 10.1039/C7AN00509A. 28513658

[ref28] Zhu G.; Sun L.; Dovichi N. J. Dynamic PH Junction Preconcentration in Capillary Electrophoresis- Electrospray Ionization-Mass Spectrometry for Proteomics Analysis. Analyst 2016, 141, 5216–5220. 10.1039/C6AN01140C. 27460877 PMC5007160

[ref29] Sun L.; Zhu G.; Zhao Y.; Yan X.; Mou S.; Dovichi N. J. Ultrasensitive and Fast Bottom-up Analysis of Femtogram Amounts of Complex Proteome Digests. Angewandte Chemie - International Edition 2013, 52 (51), 13661–13664. 10.1002/anie.201308139. 24173663 PMC3904452

[ref30] Xu T.; Han L.; George Thompson A. M.; Sun L. An Improved Capillary Isoelectric Focusing-Mass Spectrometry Method for High-Resolution Characterization of Monoclonal Antibody Charge Variants. Analytical Methods 2022, 14 (4), 383–393. 10.1039/D1AY01556G. 34939625

[ref31] Zhu G.; Sun L.; Dovichi N. J. Simplified Capillary Isoelectric Focusing with Chemical Mobilization for Intact Protein Analysis. J. Sep Sci. 2017, 40 (4), 948–953. 10.1002/jssc.201601051. 27935257

[ref32] Xu T.; Sun L. A Mini Review on Capillary Isoelectric Focusing-Mass Spectrometry for Top-Down Proteomics. Front. Chem. 2021, 9, 651757 10.3389/fchem.2021.651757. 33898392 PMC8063032

[ref33] Kou Q.; Xun L.; Liu X. TopPIC: A Software Tool for Top-down Mass Spectrometry-Based Proteoform Identification and Characterization. Bioinformatics 2016, 32 (22), 3495–3497. 10.1093/bioinformatics/btw398. 27423895 PMC5181555

[ref34] Marty M. T.; Baldwin A. J.; Marklund E. G.; Hochberg G. K. A.; Benesch J. L. P.; Robinson C. V. Bayesian Deconvolution of Mass and Ion Mobility Spectra: From Binary Interactions to Polydisperse Ensembles. Anal. Chem. 2015, 87 (8), 4370–4376. 10.1021/acs.analchem.5b00140. 25799115 PMC4594776

[ref35] Basharat A. R.; Zang Y.; Sun L.; Liu X. TopFD: A Proteoform Feature Detection Tool for Top-Down Proteomics. Anal. Chem. 2023, 95 (21), 8189–8196. 10.1021/acs.analchem.2c05244. 37196155 PMC10233584

[ref36] Lubeckyj R. A.; Basharat A. R.; Shen X.; Liu X.; Sun L. Large-Scale Qualitative and Quantitative Top-Down Proteomics Using Capillary Zone Electrophoresis-Electrospray Ionization-Tandem Mass Spectrometry with Nanograms of Proteome Samples. J. Am. Soc. Mass Spectrom. 2019, 30 (8), 1435–1445. 10.1007/s13361-019-02167-w. 30972727 PMC6675661

[ref37] Fang F.; Xu T.; Hagar H.-T. C.; Hovde S.; Kuo M.-H.; Sun L. A Pilot Study for Deciphering Post-Translational Modifications and Proteoforms of Tau Protein by Capillary Electrophoresis-Mass Spectrometry. J. Proteome Res. 2024, 23, 5085 10.1021/acs.jproteome.4c00587. 39327902 PMC11536466

[ref38] Xu T.; Han L.; Sun L. Automated Capillary Isoelectric Focusing-Mass Spectrometry with Ultrahigh Resolution for Characterizing Microheterogeneity and Isoelectric Points of Intact Protein Complexes. Anal. Chem. 2022, 94 (27), 9674–9682. 10.1021/acs.analchem.2c00975. 35766479

[ref39] Xu R.; Shen J.; Song Y.; Lu J.; Liu Y.; Cao Y.; Wang Z.; Zhang J. Exploration of the Application Potential of Serum Multi-Biomarker Model in Colorectal Cancer Screening. Sci. Rep 2024, 14 (1), 10127 10.1038/s41598-024-60867-0. 38698075 PMC11066011

[ref40] Pitkänen H. H.; Haapio M.; Saarela M.; Taskinen M. R.; Brinkman H. J.; Lassila R. Impact of Therapeutic Plasma Exchange on Intact Protein S, Apolipoproteins, and Thrombin Generation. Transfusion and Apheresis Science 2024, 63, 103918 10.1016/j.transci.2024.103918. 38555232

[ref41] Retracted: Exploration of Potential Biomarkers and Immune Landscape for Hepatoblastoma: Evidence from Machine Learning Algorithm. Evidence-Based Complementary and Alternative Medicine 2023, 2023, 9893765, 10.1155/2023/9893765. 37886423 PMC10599939

[ref42] Zhu M.; Lan Z.; Park J.; Gong S.; Wang Y.; Guo F. Regulation of CNS Pathology by Serpina3n/SERPINA3: The Knowns and the Puzzles. Neuropathol. Appl. Neurobiol. 2024, 50, e12980 10.1111/nan.12980. 38647003 PMC11131959

[ref43] Neagu A.-N.; Whitham D.; Buonanno E.; Jenkins A.; Alexa-Stratulat T.; Tamba B. I.; Darie C. C. Proteomics and Its Applications in Breast Cancer. Am. J. Cancer Res. 2021, 11, 4006. 34659875 PMC8493401

[ref44] Schreiner T. G.; Ignat B. E.; Grosu C.; Costache A. D.; Leon M. M.; Mitu F. Lipid-Derived Biomarkers as Therapeutic Targets for Chronic Coronary Syndrome and Ischemic Stroke: An Updated Narrative Review. Medicina 2024, 60, 561 10.3390/medicina60040561. 38674207 PMC11052465

[ref45] Wu B.; Yang X.; Chen F.; Song Z.; Ding X.; Wang X. Apolipoprotein E Is a Prognostic Factor for Pancreatic Cancer and Associates with Immune Infiltration. Cytokine 2024, 179, 156628 10.1016/j.cyto.2024.156628. 38704962

[ref46] Churchill R. A.; Gochanour B. R.; Scott C. G.; Vasile V. C.; Rodeheffer R. J.; Meeusen J. W.; Jaffe A. S. Association of Cardiac Biomarkers with Long-Term Cardiovascular Events in a Community Cohort. Biomarkers 2024, 29, 161 10.1080/1354750X.2024.2335245. 38666319

[ref47] Han S.; Zhang J.; Sun Y.; Liu L.; Guo L.; Zhao C.; Zhang J.; Qian Q.; Cui B.; Zhang Y. The Plasma DIA-Based Quantitative Proteomics Reveals the Pathogenic Pathways and New Biomarkers in Cervical Cancer and High Grade Squamous Intraepithelial Lesion. J. Clin Med. 2022, 11 (23), 7155 10.3390/jcm11237155. 36498728 PMC9736146

[ref48] Zhu Y.; Zhang H.; Jiang P.; Xie C.; Luo Y.; Chen J. Transcriptional and Epigenetic Alterations in the Progression of Non-Alcoholic Fatty Liver Disease and Biomarkers Helping to Diagnose Non-Alcoholic Steatohepatitis. Biomedicines 2023, 11 (3), 970 10.3390/biomedicines11030970. 36979950 PMC10046227

[ref49] Lv J. H.; Hou A. J.; Zhang S. H.; Dong J. J.; Kuang H. X.; Yang L.; Jiang H. WGCNA Combined with Machine Learning to Find Potential Biomarkers of Liver Cancer. Medicine 2023, 102 (50), e36536 10.1097/MD.0000000000036536. 38115320 PMC10727608

[ref50] Li Z.; Li S.; Luo M.; Jhong J. H.; Li W.; Yao L.; Pang Y.; Wang Z.; Wang R.; Ma R.; Yu J.; Huang Y.; Zhu X.; Cheng Q.; Feng H.; Zhang J.; Wang C.; Hsu J. B. K.; Chang W. C.; Wei F. X.; Huang H. Da; Lee T. Y. DbPTM in 2022: An Updated Database for Exploring Regulatory Networks and Functional Associations of Protein Post-Translational Modifications. Nucleic Acids Res. 2022, 50 (D1), D471–D479. 10.1093/nar/gkab1017. 34788852 PMC8728263

[ref51] Rodriguez-Muñoz A.; Motahari-Rad H.; Martin-Chaves L.; Benitez-Porres J.; Rodriguez-Capitan J.; Gonzalez-Jimenez A.; Insenser M.; Tinahones F. J.; Murri M. 92A Systematic Review of Proteomics in Obesity: Unpacking the Molecular Puzzle. Curr. Obes Rep 2024, 13, 403 10.1007/s13679-024-00561-4. 38703299 PMC11306592

[ref52] Hu C.; Zhao Z.; Dong S.; Guo Q.; Zhou Y. The Clinical Role of Combined Circulating Complement C1q and AIP for CAD with LDL-C Level below 1.8mmol/L. Lipids Health Dis 2024, 23 (1), 131 10.1186/s12944-024-02127-8. 38704561 PMC11070092

[ref53] Chrisman M.; White-Lewis S.; Lasiter S.; Chesnut S. R.; Russell C. L. Equine-Assisted Service’s Effect on Cartilage and Skeletal Biomarkers for Adults and Older Adults with Arthritis: A Pilot Study. Complement Ther Med. 2024, 82, 103047 10.1016/j.ctim.2024.103047. 38697487

[ref54] Nady A.; Reichheld S. E.; Sharpe S. Structural Studies of a Serum Amyloid A Octamer That Is Primed to Scaffold Lipid Nanodiscs. Protein Sci. 2024, 33 (5), e4983 10.1002/pro.4983. 38659173 PMC11043621

[ref55] Sołkiewicz K.; Kokot I.; Kacperczyk M.; Dymicka-Piekarska V.; Dorf J.; Kratz E. M. Serum Clusterin Concentration and Its Glycosylation Changes as Potential New Diagnostic Markers of SARS-CoV-2 Infection and Recovery Process. Int. J. Mol. Sci. 2024, 25 (8), 4198 10.3390/ijms25084198. 38673784 PMC11049940

[ref56] Chen X.-X.; Zeng M.-X.; Cai D.; Zhou H.-H.; Wang Y.-J.; Liu Z. Correlation between APOE4 Gene and Gut Microbiota in Alzheimer’s Disease. Benef Microbes 2023, 14 (4), 349–360. 10.1163/18762891-20220116. 38661357

[ref57] Hou L.; Zhang X.; Jiao Y.; Li Y.; Zhao Y.; Guan Y.; Liu Z. ATP Binding Cassette Subfamily B Member 9 (ABCB9) Is a Prognostic Indicator of Overall Survival in Ovarian Cancer. Medicine 2019, 98 (19), e15698 10.1097/MD.0000000000015698. 31083274 PMC6531167

[ref58] Li J.; Yan W.; Yuan H.; Ren F. Theacrine Enhances Autophagy and Inhibits Inflammation via Regulating SIRT3/FOXO3a/Parkin Pathway. Int. J. Rheum. Dis. 2024, 27 (2), e15085 10.1111/1756-185X.15085. 38402443

[ref59] Jiang X.; Yang L.; Gao Q.; Liu Y.; Feng X.; Ye S.; Yang Z. The Role of RAB GTPases and Its Potential in Predicting Immunotherapy Response and Prognosis in Colorectal Cancer. Front. Genet. 2022, 13, 828373 10.3389/fgene.2022.828373. 35154286 PMC8833848

[ref60] Zhao Q.; Wu Y.; Wu X.; Liu M.; Nan L. Single-Cell Transcriptome Analysis Reveals Keratinocyte Subpopulations Contributing to Psoriasis in Corneum and Granular Layer. Skin Res. Technol. 2024, 30 (2), e13572 10.1111/srt.13572. 38279596 PMC10818132

[ref61] Shavali M.; Moradi A.; Tahmaseb M.; Mohammadian K.; Ganji S. M. Circulating-Tumour DNA Methylation of HAND1 Gene: A Promising Biomarker in Early Detection of Colorectal Cancer. BMC Med. Genomics 2024, 17 (1), 117 10.1186/s12920-024-01893-9. 38689296 PMC11061902

[ref62] Xu T.; Wang Q.; Wang Q.; Sun L. Coupling High-Field Asymmetric Waveform Ion Mobility Spectrometry with Capillary Zone Electrophoresis-Tandem Mass Spectrometry for Top-Down Proteomics. Anal. Chem. 2023, 95 (25), 9497–9504. 10.1021/acs.analchem.3c00551. 37254456 PMC10540249

[ref63] Perez-Riverol Y.; Csordas A.; Bai J.; Bernal-Llinares M.; Hewapathirana S.; Kundu D. J.; Inuganti A.; Griss J.; Mayer G.; Eisenacher M.; Pérez E.; Uszkoreit J.; Pfeuffer J.; Sachsenberg T.; Yilmaz Ş.; Tiwary S.; Cox J.; Audain E.; Walzer M.; Jarnuczak A. F.; Ternent T.; Brazma A.; Vizcaíno J. A. The PRIDE Database and Related Tools and Resources in 2019: Improving Support for Quantification Data. Nucleic Acids Res. 2019, 47 (D1), D442–D450. 10.1093/nar/gky1106. 30395289 PMC6323896

