# Supplementary material for: Top-Down Proteomic Profiling of Protein Corona by High-Throughput Capillary Isoelectric Focusing-Mass Spectrometry
Source: J Am Soc Mass Spectrom. 2025 Mar 3;36(4):778–86. doi: 10.1021/jasms.4c00463 (PMC11964827; doi:10.1021/jasms.4c00463)
Supplement: Supplementary file 1 — js4c00463_si_001.pdf [file js4c00463_si_001.pdf]

Supporting Information I

Top-down proteomic profiling of protein corona by high-throughput capillary isoelectric focusing-mass spectrometry

Reyhane Tabatabaeian Nimavard,[1] Seyed Amirhossein Sadeghi,[1] Morteza Mahmoudi,[2,3] Guijie Zhu,[1,*] Liangliang Sun[1,*]

[1] Department of Chemistry, Michigan State University, 578 S Shaw Lane, East Lansing, Michigan 48824, United States

[2] Precision Health Program, Michigan State University, East Lansing, Michigan 48824, United States

[3] Department of Radiology, College of Human Medicine, Michigan State University, East Lansing, Michigan 48824, United States.

* Corresponding authors

Email: lsun@chemistry.msu.edu (L Sun); zhuguiji@msu.edu (G Zhu)

Phone: 517-353-0498

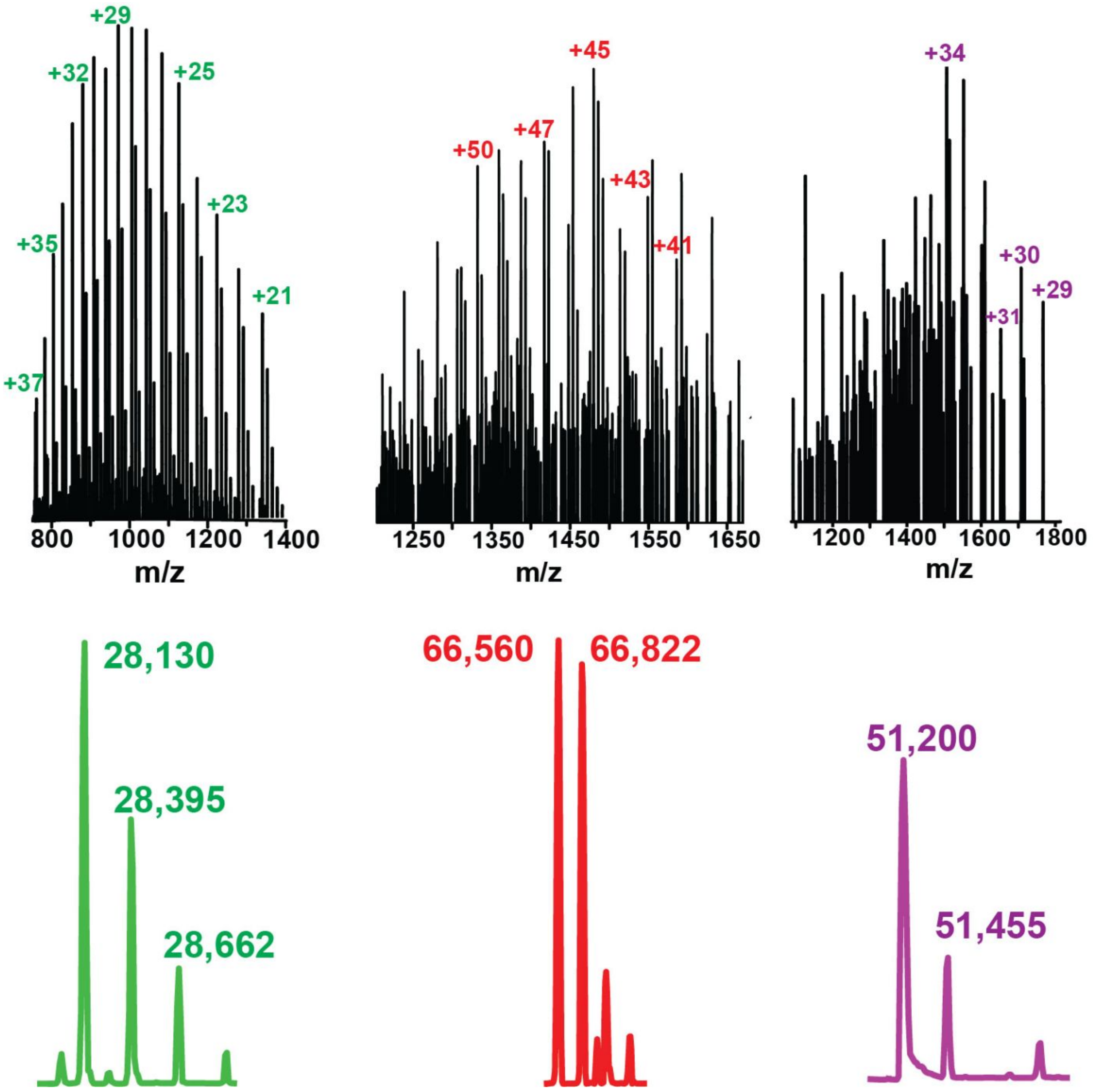

Figure S1. Mass spectra and deconvoluted mass spectra of three large proteins detected in the "Low-High" runs. Each protein has multiple proteoforms with different masses. The UniDec software was used for mass deconvolution. [1]

(1) Marty, M. T.; Baldwin, A. J.; Marklund, E. G.; Hochberg, G. K. A.; Benesch, J. L. P.; Robinson, C. V. Bayesian Deconvolution of Mass and Ion Mobility Spectra: From Binary Interactions to Polydisperse Ensembles. *Anal Chem* 2015, *87* (8), 4370–4376. https://doi.org/10.1021/acs.analchem.5b00140.
